# Increased expression of mesencephalic astrocyte-derived neurotrophic factor (MANF) contributes to synapse loss in Alzheimer’s disease

**DOI:** 10.1186/s13024-024-00771-3

**Published:** 2024-10-18

**Authors:** Yiran Zhang, Xiusheng Chen, Laiqiang Chen, Mingting Shao, Wenzhen Zhu, Tingting Xing, Tingting Guo, Qingqing Jia, Huiming Yang, Peng Yin, Xiao-Xin Yan, Jiandong Yu, Shihua Li, Xiao-Jiang Li, Su Yang

**Affiliations:** 1 Guangdong Key Laboratory of Non-Human Primate Research, Key Laboratory of CNS Regeneration (Ministry of Education), Guangdong-Hong Kong-Macau Institute of CNS Regeneration, Jinan University, Guangzhou, China; 2 State Key Laboratory of Bioactive Molecules and Druggability Assessment, Jinan University, Guangzhou, China; 3 Department of Neurology, The First Affiliated Hospital, Sun Yat-Sen University; Guangdong Provincial Key Laboratory of Diagnosis and Treatment of Major Neurological Diseases, Guangzhou, China; 4 Department of Anatomy and Neurobiology, Central South University Xiangya School of Medicine, Changsha, China; 5 Department of Neurosurgery, The First Affiliated Hospital of Bengbu Medical College, Bengbu, China

**Keywords:** Alzheimer’s disease, MANF, Synapse, ER stress; ELAVL2

## Abstract

**Background:**

The activation of endoplasmic reticulum (ER) stress is an early pathological hallmark of Alzheimer’s disease (AD) brain, but how ER stress contributes to the onset and development of AD remains poorly characterized. Mesencephalic astrocyte-derived neurotrophic factor (MANF) is a non-canonical neurotrophic factor and an ER stress inducible protein. Previous studies reported that MANF is increased in the brains of both pre-symptomatic and symptomatic AD patients, but the consequence of the early rise in MANF protein is unknown.

**Methods:**

We examined the expression of MANF in the brain of AD mouse models at different pathological stages. Through behavioral, electrophysiological, and neuropathological analyses, we assessed the level of synaptic dysfunctions in the MANF transgenic mouse model which overexpresses MANF in the brain and in wild type (WT) mice with MANF overexpression in the hippocampus. Using proteomic and transcriptomic screening, we identified and validated the molecular mechanism underlying the effects of MANF on synaptic function.

**Results:**

We found that increased expression of MANF correlates with synapse loss in the hippocampus of AD mice. The ectopic expression of MANF in mice via transgenic or viral approaches causes synapse loss and defects in learning and memory. We also identified that MANF interacts with ELAV like RNA-binding protein 2 (ELAVL2) and affects its binding to RNA transcripts that are involved in synaptic functions. Increasing or decreasing MANF expression in the hippocampus of AD mice exacerbates or ameliorates the behavioral deficits and synaptic pathology, respectively.

**Conclusions:**

Our study established MANF as a mechanistic link between ER stress and synapse loss in AD and hinted at MANF as a therapeutic target in AD treatment.

**Supplementary Information:**

The online version contains supplementary material available at 10.1186/s13024-024-00771-3.

## Background

Alzheimer's disease (AD) is the most prevalent type of dementia worldwide, characterized by a decreased synaptic density in the hippocampus and cortex. The loss of synapses strongly correlates with cognitive decline during AD pathogenesis [1–3]. Two typical hallmarks of AD are the accumulation of senile plaques composed of amyloid beta (Aβ) and neurofibrillary tangles composed of hyperphosphorylated tau [4, 5]. The presence of misfolded proteins triggers disturbances in protein homeostasis, which affects the function of endoplasmic reticulum (ER) and cause ER stress. ER stress activates the unfolded protein response (UPR), which is an adaptive reaction that promotes the clearance of misfolded proteins, but can also lead to cell death if the stress is irreversible [6]. Emerging evidence suggests that ER stress contributes to the pathogenesis of AD, as the expression of multiple ER stress markers is increased during the early stage of AD in both patient brains and mouse models [7–9]. Nonetheless, how ER stress causes synaptic impairments in AD remains poorly understood.

Mesencephalic astrocyte-derived neurotrophic factor (MANF) is considered a non-canonical neurotrophic factor, given its distinct protein structure and functional mechanisms [10–12]. Extracellularly, the administration of recombinant MANF protein affords neuroprotection in multiple disease conditions, including Parkinson’s disease (PD), ischemic stroke and retinal degeneration [13–16], although the transmembrane receptor that mediates such protective effects has not been clearly defined [17]. Intracellularly, MANF is enriched in the ER and its expression is stimulated upon ER stress [18–20]. MANF alleviates ER stress, potentially via binding to the UPR sensor IRE1α and the ER chaperone glucose-regulated protein 78 (GRP78) [21–24]. However, these studies are mainly based on in vitro models of acute ER stress. The consequence of sustained MANF upregulation in vivo is unknown.

Previous studies indicate that MANF expression is increased in the brain of AD patients and mice [25, 26], but the exact role of increased MANF in AD pathogenesis remains unexplored. In the present study, we systematically examined the expression of MANF in AD mouse brain and found that its upregulation correlates with synaptic loss in the hippocampus. This result indicates that sustained MANF upregulation leads to synaptic impairments. Coincidentally, overexpression of MANF in the brain of a MANF transgenic mouse model [27, 28] leads to learning and memory deficits and synaptic dysfunctions. We further identified the RNA-binding protein ELAVL2 (ELAV like RNA-binding protein 2, alternatively named HuB) as a MANF interacting protein, whose RNA-binding affinity is disrupted upon MANF overexpression, leading to altered expression of transcripts related to synaptic functions. Finally, modulating MANF expression had a pronounced effect on both behavioral outcomes and synaptic pathology in AD mice: increasing MANF exacerbated these aspects, while reducing MANF ameliorated them. Our results demonstrate that sustained upregulation of MANF contributes to synaptic loss in AD mice by affecting ELAVL2 mediated post-transcriptional program, and indicate that lowering MANF expression could be exploited as a potential therapeutic approach to alleviate the synaptic pathology in AD.

## Methods

### Human brain samples

Postmortem human brains were banked through a willed body donation program [29], with the donor’s clinical records prior to and/or during the last hospitalization obtained whenever available. All the brains were histopathologically processed according to the Standard Brain Banking Protocol set by China Brain Bank Consortium [30]. The brains were dissected and preserved fresh-frozen at -70 °C. Three AD cases (aged over 65 years) and three age-matched control cases (aged over 65 years) were included in the analyses. The stages of Aβ and tau pathology were assessed according to the established standards [31, 32]. The age-matched control cases had no known clinical history of dementia. The patient information is provided in Table 1.

**Table 1:** Demographic information of the brain donors. Braak neurofibrillary tangle (NFT) stage was determined by immunostaining with the AT8 antibody. Thal Aβ phase was determined by immunostaining with the 6E10 antibody

Case #	Group	Age (years)	Sex	Clinical diagnosis and cause of death	Postmortem delay(hours)	Braak NFT stage	Thal Aβ phase
1	Control	79	M	Stroke	2	0	0
2	Control	76	M	Myocardial infarction	7	0	0
3	Control	81	M	Cardiovascular disease	6	II	0
4	AD	86	M	Coronary heart disease and dementia	6	III	3
5	AD	83	F	Multiple system failure	12	IV	5
6	AD	89	M	Multiple system failure	8	III	2

### Animals

Wild-type C57BL6J mice were purchased from Guangdong Medical Laboratory Animal Center (Guangzhou, China). MANF transgenic mice were described in our previous study [28] and generated by Cyagen Biosciences (Guangzhou, China). 5xFAD mice were from the Jackson Laboratory (hemizygous, strain #034848-JAX). APP/PS1 mice (C57BL6J background) were from Cavens-Biogle (Suzhou, China). All the mice were maintained in a 12-h light/dark cycle in the Division of Animal Resources of Jinan University. Both male and female mice were used in every experiment of this study. The primers used for genotyping are listed as follows. For MANF transgenic mice, forward: 5’-ATT GAC CTG AGC ACA GTG GAC CTG-3’; reverse: 5’-GTC ACT GTC ACC TTG TAC TCT GG-3’. For 5xFAD mice, common: 5’-ACC CCC ATG TCA GAG TTC CT-3’; WT: 5’-TAT ACA ACC TTG GGG GAT GG-3’; mutant: 5’-CGG GCC TCT TCG CTA TTA C-3’. For APP/PS1 mice, common: 5’-GGA TCT CTG AGG GGT CCA GT-3’; WT: 5’-GTG TGA TCC ATT CCA TCA GC-3’; mutant: 5’-ATG GTA GAG TAA GCG AGA ACA CG-3’.

### Antibodies

The primary antibodies used in the study are listed as follows: MANF (LSBio, B2688; Abcam, ab67271; EM572, self-made), APP (Biolegend, 803001), amyloid beta (Abcam, ab126649), NeuN (Abcam, ab177487; Millipore, MAB377), β-tubulin (Proteintech, 10094–1-AP), PSD95 (Abcam, ab238135; GeneTex, GTX133091), synaptophysin (Santa Cruz Biotechnology, sc-12737), HA tag (Abcam, ab9110), GFAP (Sigma, G3893; Abcam, ab7260), IBA1 (Abcam, ab178846), GRP78 (Zen-bioscience, 200310-4F11), GST tag (Invitrogen, MA4-004), His tag (Cell signaling, 9715), Histone H3 (Cell signaling, 2365), Vinculin (Millipore, MAB3574), Cas9 (Sigma, MAC133), GFP (Emarbio, EM33012), ELAVL2 (Proteintech, 14008–1-AP, 67097–1-Ig), GRIA1 (Proteintech, 67642–1-Ig), GRIN2A (Proteintech, 28525–1-AP).

### Mouse behavioral tests

For all the behavioral tests, the mice were randomly assigned to the experimental groups, and the tests were performed by a person blinded to the experimental groups. Individual data points that deviated by more than three standard deviations from the mean were classified as outliers and were excluded from the data analysis.

Novel object recognition was performed according to a published protocol [33]. Briefly, on Day 1, the mice were placed in a white plastic box measuring 33 × 33 × 20 cm for habituation. On Day 2, two identical objects (wooden circular cones) were placed in the box and the mice were allowed to explore the objects for 10 min. On Day 3, one old object was replaced with one new object (wooden cube), and the time the mice spent exploring the old and new objects was recorded using two stop watches.

For fear conditioning, the mice were placed in the apparatus (Coulbourn) composed of Plexiglas with a metal shock grid floor. Three conditioned stimulus (CS)—unconditioned stimulus (US) pairings were presented with a 1-min inter-trial interval. The CS consists of a 20-s 85-db tone, and the US consists of a 2-s 0.5-mA foot shock. The shock was delivered via a Precision Animal Shocker (Colbourn) connected to each fear conditioning chamber. On Day 2, the mice were placed in the same chamber used on Day 1, and the amount of freezing will be recorded via a camera and the software provided by Colbourn. On Day 3, the mice were exposed to the CS in a novel compartment. Following a 2-min habituation period, the 85-db tone was presented for 6 min, and the amount of freezing behavior was recorded.

For elevated plus maze, the mice were transferred to the testing room 30 min prior to the test for habituation. The mice were individually placed in the crossover of the open and closed arm, facing the open arm. The movement of the mice was recorded by a video camera mounted above the maze for 5 min. The recorded videos were analyzed using the TopScan behavior analyzing system.

For open field, an automated system was used (Photobeam activity system, San Diego Instruments). One day before the test, the mice were transferred to the test room in their home cages to acclimate. On the test day, each mouse was placed in a separate test chamber and allowed to explore the chamber freely for 30 min. The locomotion of the mice in the chamber was automatically recorded as infrared beam breaks.

Rewarded alternation T-maze was performed according to a published protocol [34]. Prior to the test, the mice were subjected to food restriction and were placed in the testing room for 30 min for habituation. In the training phase, individual mouse was placed in the starting arm. One biscuit was placed at the end of one goal arm, and the other goal arm was closed. After the mouse entered the goal arm and consumed the biscuit, these two goal arms were swapped alternately. Three rounds of training were conducted for three days. In the testing phase, one biscuit was placed at the end of one goal arm with the other one closed. Individual mouse was placed in the starting arm and allowed to enter the goal arm to consume the biscuit. For the first trial, one biscuit was placed in the other goal arm while keeping both arms open. If the mouse entered the arm with the biscuit, it was recorded as a correct trial and the next trial ensued with the goal arms swapped. If the mouse entered the arm without the biscuit, it was recorded as an incorrect and the biscuit remained in the same arm in the next trial. The trial was repeated ten times, and the correct rate of each mouse was calculated.

### Western blotting

The brain tissues were lysed in ice-cold RIPA buffer containing a protease inhibitor cocktail (Mei5bio, MF182-plus-10) and phosphatase inhibitors (Sigma, S7920 and S6508). The lysates were sonicated for 5 s × 6 times and centrifuged at 12,000 g for 15 min at 4 °C. The supernatants were collected and subjected to SDS–PAGE. The proteins in the gel were transferred to a nitrocellulose membrane. The blots were blocked with 5% milk/TBST for 1 h at room temperature and incubated with selected primary antibodies in 3% BSA/TBST overnight at 4 °C. After three washes in PBS, the blots were incubated with HRP-conjugated secondary antibodies in 5% milk/TBST for 1 h at room temperature. The blots were then washed three times in TBST and developed using ECL Prime (Millipore, WBKLS0500). The images were acquired digitally using Clinx ChemiScope 6300.

### Immunofluorescent staining

The mice were intracardially perfused with warm 0.9% saline solution, followed by 4% ice-cold paraformaldehyde (PFA) in 0.1 M PB solution. The brains of the mice were separated and fixed overnight in 4% PFA solution and transferred to 15% sucrose for 24 h, and to 30% sucrose for another 24 h. The brains were embedded in the OCT solution (Sakura, 4583) and sectioned at 30 μm in a cryostat (Thermo Fisher). The slices were blocked with 3% bovine serum albumin in PBS with 0.2% Triton X-100 (Sigma, X100) for 30 min at room temperature. The slices were then incubated with selected primary antibodies (buffered in PBS with 0.2% Triton X-100) at 4 °C overnight. On the following day, the slices were washed with PBS three times and incubated with secondary antibodies and nuclear dye DAPI (buffered in PBS with 0.2% Triton X-100) for 1 h at room temperature. Images were acquired using an Olympus FV3000 confocal laser scanning microscope.

The ImageJ software (Ver 1.54d) was used for the quantification of staining intensity or area. The image type was converted to 8-bit grayscale. The “Threshold” function was used to subtract background, and the “Measure” function was used to quantify the staining intensity or area. For the quantification of PSD95 and synaptophysin co-localized puncta, high-resolution images were captured using an Olympus FV3000 confocal laser scanning microscope equipped with a 100 × objective lens. The ImageJ plugin “ComDet” (v.0.5.5) was used with advanced scaling settings. The images were processed by navigating to “Image-Color-Make composite”, followed by appropriate zooming. A 10 μm × 10 μm rectangle was framed using the "Rectangle" tool. Subsequently, the "Detect particles" function was used with parameters set as follows: a maximum distance of 6 pixels between colocalized spots, an approximate particle size of 3 pixels, and an intensity threshold (in standard deviations) ranging from 3 to 5 to encompass all staining signals. The colocalized signals were automatically counted. To the quantify the co-staining area of IBA1 and synaptophysin, both images were imported into the "JaCoP" plugin of ImageJ. The “Threshold” function was used to subtract background and the Manders' coefficients were calculated. The M2 value, which represents the overlapping area of IBA1 and synaptophysin, was used for statistical analysis.

### Immunohistochemistry

The brain slices were blocked in blocking buffer (3% BSA/2% goat serum/0.1% Triton X-100 in 1 X PBS) for 1 h and incubated with selected primary antibodies in the blocking buffer at 4 °C overnight. After washing with PBS three times, the brain slices were incubated with secondary antibodies for 10 min and developed with the Mouse and Rabbit Specific HRP/DAB (ABC) Detection IHC kit (Abcam, ab64264) following the manufacturer’s protocol. Images were acquired using a Zeiss AX10 Axio microscope. The ImageJ software was used for the quantification of staining intensity. The image type was converted to 8-bit grayscale. The “Threshold” function was used to subtract background, and the “Measure” function was used to quantify the staining intensity.

### Enzyme-linked immunosorbent assay (ELISA)

The ELISA kit (Invitrogen, KHB3441) specific to Aβ42 was used according to the manufacturer’s instructions. Mouse brain tissues were homogenized and sonicated in PBS containing a protease inhibitor cocktail (Mei5bio, MF182-plus-10) and phosphatase inhibitors (Sigma, S7920 and S6508), followed by centrifugation at 12,000 g for 15 min at 4 °C. The concentration of the samples was adjusted to 1 μg/μl and diluted 1:500 before use. The standard curve was calculated using the ELISACalc software. Aβ42 concentrations in the samples were determined by fitting the standard curve.

### Electrophysiology

The brain slices of 250 μm were prepared in pre-cooled NMDG solution containing 93 mM NMDG, 2.5 mM KCl,1.2 mM NaH2PO4, 25 mM D-glucose, 30 mM NaHCO3, 20 mM HEPES, 5 mM sodium ascorbate, 2 mM Thiourea, 3 mM sodium pyruvate, 10 mM MgSO4 and 0.5 mM CaCl_2_. The solution was titrated with HCl to pH 7.25 at an osmotic pressure of 300 ~ 310 mOsm. The prepared brain slices were incubated in the NMDG solution with oxygen (95% O_2_ and 5% CO_2_) and kept at 32 ± 1 °C for 1 h. The brain slices were placed in the recording chamber with artificial cerebrospinal fluid (aCSF) containing 126 mM NaCl, 2.5 mM KCl, 1.25 mM NaH_2_PO_4_, 26 mM NaHCO_3_, 2 mM MgCl_2_, 2 mM CaCl_2_, and 10 mM glucose, and recorded with patch pipettes (6–8 MΩ) filled with internal solutions. Gluconate internal electrode fluid was used for recording (126 mM potassium gluconate, 4 mM KCl, 10 mM HEPES, 4 mM Mg-ATP, 0.5 mM Na-GTP, and 10 mM creatine phosphate). Whole-cell recordings of hippocampal CA1 pyramidal cells were performed via IR-DIC visualization under a Nikon Eclipse FN-1 microscope. The cells were recorded with a holding potential of − 70 mV. The data were collected using a Multiclamp 700B amplifier (Molecular Devices), with low pass filtered at 3 kHz and sampled at 10 kHz. The data were analyzed with Clampfit 10.4.

### Golgi staining

The Golgi–Cox Impregnation & Staining System was used according to the manufacturer’s instruction (FD Rapid GolgiStainTM kit, FD NeuroTechnologies). After impregnation, brain sections (100 μm) were obtained using a cryostat and mounted to gelatin-coated glass slides. Sequential images were taken with an Olympus FV3000 microscope under a 63 × oil objective using the z-stack function at 10 μm intervals. The density of apical densities from the CA1 region of the hippocampus was measured by manually counting the number of spines along 100 μm of the dendrites using the “Multi-point” tool in the ImageJ software. Each group contained three mice and eight segments from each mouse were randomly selected for quantification.

### Virus packaging and stereotaxic injection

The AAV-MANF, AAV-GFP, AAV-Ctrl-gRNA, AAV-Manf-gRNA, and AAV-Cas9 viral vectors were generated and described in our previous study [27]. These viral vectors were sent to PackGene Biotech Inc for packaging (AAV9 serotype, 1 × 10^13^ vg/ml). The method for stereotaxic injection was adopted from our previous study [27]. The mice were anesthetized with 1.5% isoflurane inhalation and stabilized in a stereotaxic instrument (RWD, 69100). The hair around the surgical site was removed using a disinfected scissor and the skin was sterilized with 70% alcohol. The injection site was determined according to the distance from the bregma: anterior–posterior =  − 2.5 mm, medial–lateral =  ± 2.0 mm, dorsal–ventral =—2.0 mm. A small hole was drilled on the skull, and a 30-gauge Hamilton microsyringe was used to deliver the virus at a speed of 200 nl per minutes (bilateral injection, 1 μl in each side). After surgery, the mice were placed on a heated blanket to recover from the anesthetic.

### RNA sequencing

The mouse hippocampal tissues were dissected and sent to Novogene Co, Ltd for RNA extraction, library construction and sequencing. The raw data were quantified using Salmon software (Ver. 1.9.0) with the mapping-based mode. The differentially expressed genes (DEGs) were analyzed using the edgeR package (Ver 3.1.6) with *P* value < 0.05 and |log2Fold Change|> 0.5. Correlation map was plotted using corrplot (Ver 0.92) package. To explore the related GO and KEGG pathways, the clusterProfiler (Ver 4.0.2) package was used for the up-regulated or down-regulated genes with *P* value < 0.05. The ComplexHeamap package (Ver 2.16.0) was used to draw heatmap plot. All analyses were performed using the R (Ver 4.3.0) and the Rstudio (Ver 2023.09.1 + 494) software.

### Recombinant protein production

The pET-28a vector expressing His tagged MANF protein and the method for purification of the recombinant protein was described in our previous study [27]. Full-length ELAVL2 and ELAVL2 fragments were amplified from the mouse cDNA library and cloned into the pGEX-4T-1 vector. Primers used are listed as follows. Full length ELAVL2, forward: 5’-TAG GAT CCA TGG CAG TCA GAC TGT GTG ATG-3’; reverse: 5’-TAG AAT TCT TAG GCT TTG TGC GTT TTG TTT G-3’. Fragment RRM1, forward: 5’-TAG GAT CCA TGG CAG TCA GAC TGT GTG ATG-3’; reverse: 5’-TAG AAT TCT GAG GCT GAG CTT GGG CGA G-3’. Fragment RRM2, forward: 5’-TAG GAT CCA TCA GAG ATG CAA ACT TAT ACG TC-3’; reverse: 5’-TAG AAT TCG GCC TGA TTG GTT TTT TGG CTT G-3’. Fragment RRM3, forward: 5’-TAG GAT CCT CCC AGC TGT ACC AGT CTC CA-3’; reverse: 5’-TAG AAT TCT TAG GCT TTG TGC GTT TTG TTT G-3’.

For protein production, the vectors were transformed into BL21(DE3) competent cells, and the cells were induced by IPTG for 1 h at 37 °C. The cells were lysed in lysis buffer (1% Triton X-100 and 1:500 PMSF in 1 × PBS) by sonication, and then mixed with glutathione beads (Sigma) at 4 °C overnight. The beads were washed (0.5% Triton X-100 and 1:500 PMSF in 1 × PBS) three times, re-suspended in 1 × PBS, and stored at 4 °C.

### Immunoprecipitation and in vitro binding assay

The cell or brain lysates were homogenized in NP-40 buffer (50 mM NaCl, 50 mM Tris–HCl pH8.0, 0.1% Triton X-100, 0.5% NP-40), and 300 μg of protein was used for one experiment. The protein mixture was first pre-cleared with protein A-agarose (Sigma) for 1 h and then incubated with the primary antibody overnight. The next day, protein A-agarose was added to the mixture and incubated for 1 h. The precipitated antibody-protein complexes were collected for western blotting.

For in vitro binding assay, His-MANF recombinant protein was mixed with glutathione beads conjugated with full-length ELAVL2 or ELAVL2 fragments in 500 μl of NP40 buffer and kept at 4 °C overnight. On the next day, the beads were washed with NP40 buffer three times, and the precipitated beads–protein complexes were used for western blotting.

### Quantitative real-time PCR

The RNA was extracted from the brain tissues using the TRIzol reagent (Invitrogen). Equal amount of RNA was used for cDNA synthesis, using PrimeScript RT Reagent Kit with gDNA Eraser (Takara, RR047A). For quantitative real-time PCR, QuantiNova™ SYBR Green PCR kit (QIAGEN, 208,052) was used. The reaction was performed in CFX Connect Real-Time System (Bio-Rad). Primers used are listed as follows. *Gapdh*, forward: 5’-ATC ACT GCC ACC CAG AAG ACT GT-3’; reverse: 5’-ATG ACC TTG CCC ACA GCC TTG G-3’. *Adcy8*, forward: 5’-GGC TTC CTA CAC CTT GAC TGT-3’; reverse: 5’-ATG ACC CCT CGG TAG CTG TAT-3’. *Casp3*, forward: 5’-CTC GCT CTG GTA CGG ATG TG-3’; reverse: 5’-TCC CAT AAA TGA CCC CTT CAT CA-3’. *Htrb1*, forward: 5’-CGC CGA CGG CTA CAT TTA C-3’; reverse: 5’-AGC GAT TAC AAA GGC GTT GGA-3’. *Cyp2j9*, forward: 5’-ATG CGC CTT CCT TTC GTG G-3’; reverse: 5’-CCA GGC TTA GAA CAT TCC CGT A-3’. *Plk2*, forward: 5’-GAC TAC TGC ACC ATA AGC ATG T-3’; reverse: 5’-CTT CTG GCT CTG TCA ACA CCT-3’. *Hrh1*, forward: 5’-CAG ACC TGA TTG TAG GGG CAG-3’; reverse: 5’-CAT AGA GAG CCA AAA GAG GCA G-3’. *Bdnf*, forward: 5’-TCA TAC TTC GGT TGC ATG AAG G-3’; reverse: 5’-ACA CCT GGG TAG GCC AAG TT-3’. *Map3k1*, forward: 5’-TAA ATA CCG GGT GTT TAT TGG GC-3’; reverse: 5’-TTT TCT CCA TAA CAT GGG GTC AG-3’. *Nup93*, forward: 5’-CGT TCC CGT ACC CTC ACA C-3’; reverse: 5’-AAG TCC CCT TGA CCC GAG AA-3’. *Cyp2j9*, forward: 5’-ATG CGC CTT CCT TTC GTG G-3’; reverse: 5’-CCA GGC TTA GAA CAT TCC CGT A-3’. *Gng13*, forward: 5’-AGA GCC TCA AGT ACC AAC TGG-3’; reverse: 5’-GGG TCC TTG GGG ATT CCA T-3’.

### RNA Immunoprecipitation

The RiboCluster Profiler RIP-Assay kit (MBL, RN1001) was used according to the manufacturer’s instructions. The mouse brains were homogenized with a glass grinder in 1 mL of lysis buffer and incubated on rotation for 30 min at 4 °C. The lysate was centrifuged at 12,000 × *g* for 5 min at 4 °C. The supernatant was transferred to a tube containing the primary antibody-conjugated protein A magnetic beads (Invitrogen, 88846) and incubated with rotation for 3 h at 4 °C. The beads-RNP complexes were then washed and subjected to RNA isolation. All the operations were conducted on an RNA-free workbench.

### Subcellular fractionation

The mouse brains were homogenized using a glass grinder in the buffer containing 0.32 M sucrose, 4 mM HEPES, and adjusted to pH 7.5. The homogenate was filtered through a 40 μm cell strainer (Biologix, 15–1040) and centrifuged at 800 × *g* for 10 min. The resulting pellet (P1) and supernatant (S1) were carefully separated. The P1 fraction was resuspended in 0.32 M sucrose and centrifuged again at 800 × *g* for 10 min to obtain the nuclear fraction, whereas the supernatant was discarded. The S1 fraction was subjected to centrifugation at 800 × *g* and the supernatant was collected as the cytoplasmic fraction.

### Statistical analysis

The data were analyzed using the Prism 9 software (GraphPad). For comparisons between two groups, two-tailed student t-test was used. For three or more groups, one-way ANOVA with Tukey's multiple comparisons test was used. All experiments were repeated at least three times, and the quantification was presented as mean ± SEM. A *P* value less than 0.05 was considered statistically significant.

## Results

### The expression of MANF is increased in the brain of AD mouse models and patients

**Fig. 1:**
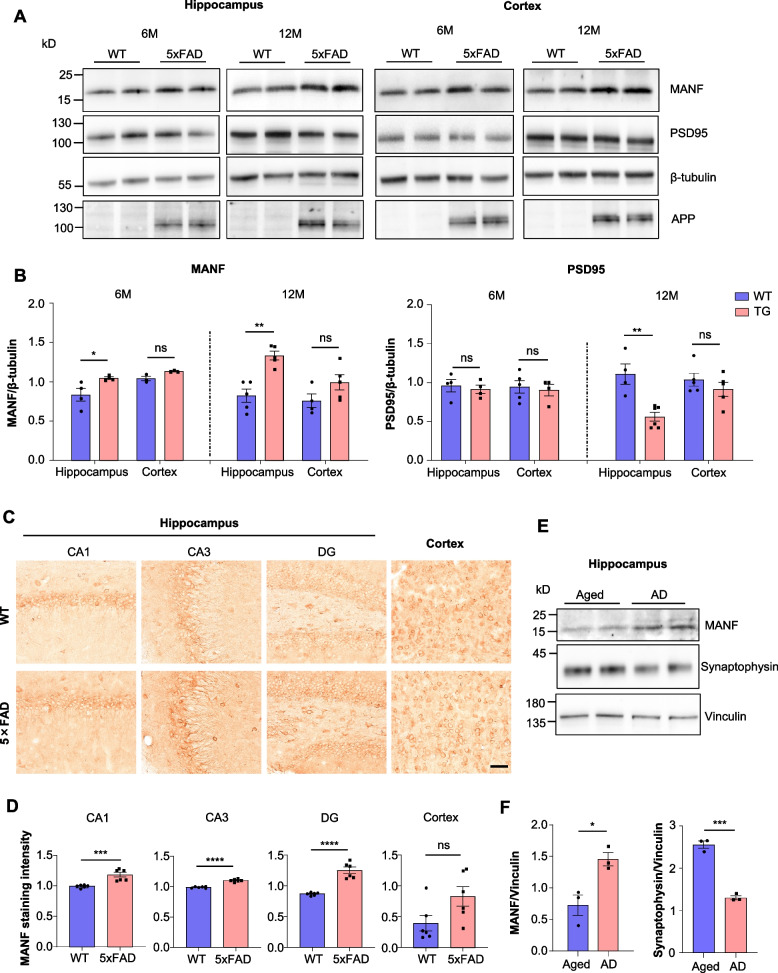
Increased expression of MANF correlates with synaptic pathology in the hippocampus of AD brain. **A** Representative western blotting images of MANF and PSD95 expression in the hippocampus and cortex of 5xFAD mice at 6 and 12 months of age. APP indicated mouse genotypes, and β-tubulin was used as a loading control. **B** Quantification of western blotting results in Fig. 1A (*n* = 3–6; two-tailed student t-test; MANF 6 M, hippocampus, *P* = 0.0417, cortex, *P* = 0.2406; MANF 12 M, hippocampus, *P* = 0.0010, cortex, *P* = 0.1212; PSD95 6 M, hippocampus, *P* = 0.6321, cortex, *P* = 0.7167; PSD95 12 M, hippocampus,* P* = 0.0023, cortex, *P* = 0.3475). **C** Immunohistochemistry images showed that MANF expression is increased in CA1, CA3 and dentate gyrus (DG) of the hippocampus from 5xFAD mice (scale bar: 50 μm). **D** Quantification of MANF staining intensity in Fig. 1C (*n* = 5–6; two-tailed student t-test; CA1, *P* = 0.0009; CA3, *P* < 0.0001; DG, *P* < 0.0001; cortex, *P* = 0.0574). **E** Representative western blotting images of MANF and synaptophysin expression in the hippocampus of AD patients and age-matched non-AD controls. **F** Quantification of western blotting results in Fig. 1E (*n* = 3; two-tailed student t-test; MANF, *P* = 0.0198; synaptophysin, *P* = 0.0002). Ns, non-significant, * *P* < 0.05, ** *P* < 0.01, *** *P* < 0.001, **** *P* < 0.001. Data are represented as mean ± SEM

First, we examined the neuropathology and MANF expression in the brain of 5xFAD mice at different ages. At the age of 6 months, the 5xFAD mice were at an early symptomatic phase, as the expression of the postsynaptic protein PSD95 in the cortex and hippocampus was comparable between the 5xFAD mice and WT littermates (Fig. 1A, B), suggesting no obvious synaptic loss. Immunohistochemical staining showed Aβ deposition in the cortex and hippocampus (Fig. S1A). In addition, reactive astrocyte marker GFAP and microglia marker IBA1 were significantly upregulated (Fig. S1B, C) in the hippocampus, which is indicative of glial activation. At this age, the expression of MANF showed a modest but significant increase in the hippocampus of the 5xFAD mice (Fig. 1A, B). At the age of 12 months, abundant Aβ plaques were found in the cortex and hippocampus by immunohistochemistry (Fig. S1A), and the expression of GFAP and IBA1 are further elevated (Fig. S1B, C). However, severe synaptic loss was only found in the hippocampus but not the cortex, indicated by the significant reduction of PSD95 specifically in the hippocampus (Fig. 1A, B). Accordingly, we found a more pronounced increase of MANF expression in the hippocampus, but not in the cortex (Fig. 1A, B). This result was further corroborated by immunohistochemistry, as the staining intensity of MANF was significantly elevated in different areas of the hippocampus, but not in the cortex from 12-month-old 5xFAD mice (Fig. 1C, D). In addition, linear regression analysis indicated a negative correlation between the expression level of MANF and PSD95, and a positive correlation between MANF and Aβ load in the hippocampus of 5xFAD mice (Fig. S1D, E).

MANF is enriched in the ER but also has functions on the plasma membrane and in the cytosol [35, 36]. Accordingly, double immunofluorescent staining revealed a partial overlap between MANF and ER marker GRP78 in the mouse brain (Fig. S2A). MANF was initially discovered from the culture medium of one astrocyte cell line [37], hence getting its name. However, multiple studies have indicated that endogenous MANF is mainly expressed in the neurons of WT mice [27, 38, 39]. By double immunofluorescent staining, we found that endogenous MANF was also more abundant in the neurons than in the astrocytes of 5xFAD mice (Fig. S2B).

We examined the brain of another AD mouse model (APP/PS1) at the age of 12 months, which again showed significantly increased MANF expression in the hippocampus (Fig. S2C, D). In addition, we collected the hippocampus tissues from post-mortem AD patients and age-matched non-AD controls. Western blotting analysis showed that the presynaptic protein synaptophysin was significantly reduced in AD samples, whereas the level of MANF was about one-fold higher in AD samples (Fig. 1E, F). Together, these results indicate that the induction of MANF expression is associated with the synaptic pathology in AD hippocampus.

### MANF overexpression leads to learning and memory impairments in mice

**Fig. 2:**
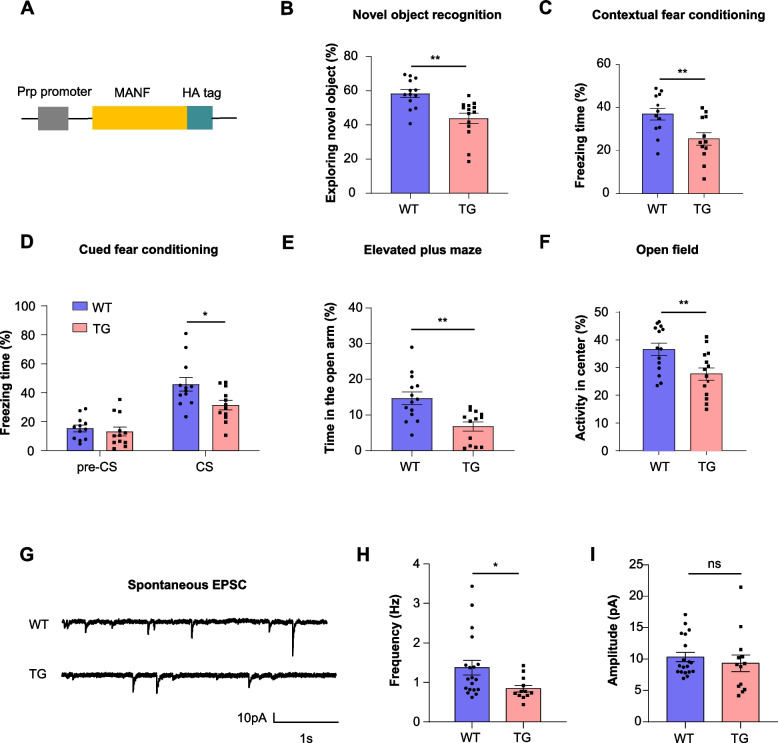
MANF transgenic mice exhibit behavioral and electrophysiological deficits. **A** Schematic representation of the transgenic MANF construct. **B** Novel object recognition test was performed on wild type (WT) and MANF transgenic (TG) mice at the age of 6 months. The percentage of time spent exploring the novel object was calculated (*n* = 13–14; two-tailed student t-test; *P* = 0.0010). **C** Contextual fear conditioning test was on performed on WT and MANF TG mice at the age of 6 months. The percentage of freezing time in the same context was calculated (*n* = 12; two-tailed student t-test; *P* = 0.0095). **D** Cued fear conditioning test was on performed on WT and MANF TG mice at the age of 6 months. The percentage of freezing time before (pre-CS) and after conditioned stimulus (CS) was calculated (*n* = 10–12; two-tailed student t-test; *P* = 0.0208). **E** Elevated plus maze was performed on WT and MANF TG mice at the age of 6 months. The percentage of time spent in the open arm was calculated (*n* = 14; two-tailed student t test; *P* = 0.0013). **F** Open field test was performed on WT and MANF TG mice at the age of 6 months. The percentage of time spent in the center was calculated (*n* = 14; two-tailed student t-test; *P* = 0.0090). **G** Representative recordings of excitatory postsynaptic potential (EPSC) were obtained from the hippocampal neurons of WT and MANF TG mice at the age of 6 months. **H**-**I** Quantification of EPSC frequency and amplitude (*n* = 12–19 from three mice; two-tailed student t-test; frequency, *P* = 0.0343; amplitude, *P* = 0.4834). Ns, non-significant, * *P* < 0.05, ** *P* < 0.01, *** *P* < 0.001. Data are represented as mean ± SEM

AD patients are characterized by cognitive decline [40] and neuropsychiatric symptoms, such as anxiety, are commonly observed [41, 42]. The defects in cognition and anxiety have been recapitulated in AD mouse models, which can be assessed by a battery of behavioral tests [43–46]. We wonder whether the increased expression of MANF in the brain is associated with these AD-like phenotypes. We previously established a MANF transgenic mouse model in which exogenous MANF is driven by the neuron-specific prion promoter (Fig. 2A), so that transgenic MANF is only detected in the neurons but not in the astrocytes [27, 28]. Novel object recognition and fear conditioning tests revealed that MANF transgenic mice at the age of 6 months had impaired cognitive performances compared with age-matched littermates (Fig. 2B-D). We also performed elevated plus maze and open field tests and found significantly heightened anxiety in the MANF transgenic mice (Fig. 2E, F).

Given the hippocampus is the brain region that is closely linked to the above behavioral tests, we performed whole-cell patch clamp to record the electrophysiology of neurons in the hippocampal CA1 region of 6-month-old MANF transgenic mice. We found that the frequency of spontaneous excitatory postsynaptic current (EPSC) in the MANF transgenic mice was significantly lower than that in WT mice (Fig. 2G, H), while the amplitude was unchanged (Fig. 2I), which indicates a loss of synaptic number. In agreement with the electrophysiological changes, the expression of glutamate receptors was significantly reduced in the hippocampus of MANF transgenic mice (Fig. S3A, B).

### MANF overexpression causes synapse loss in the hippocampus

**Fig. 3:**
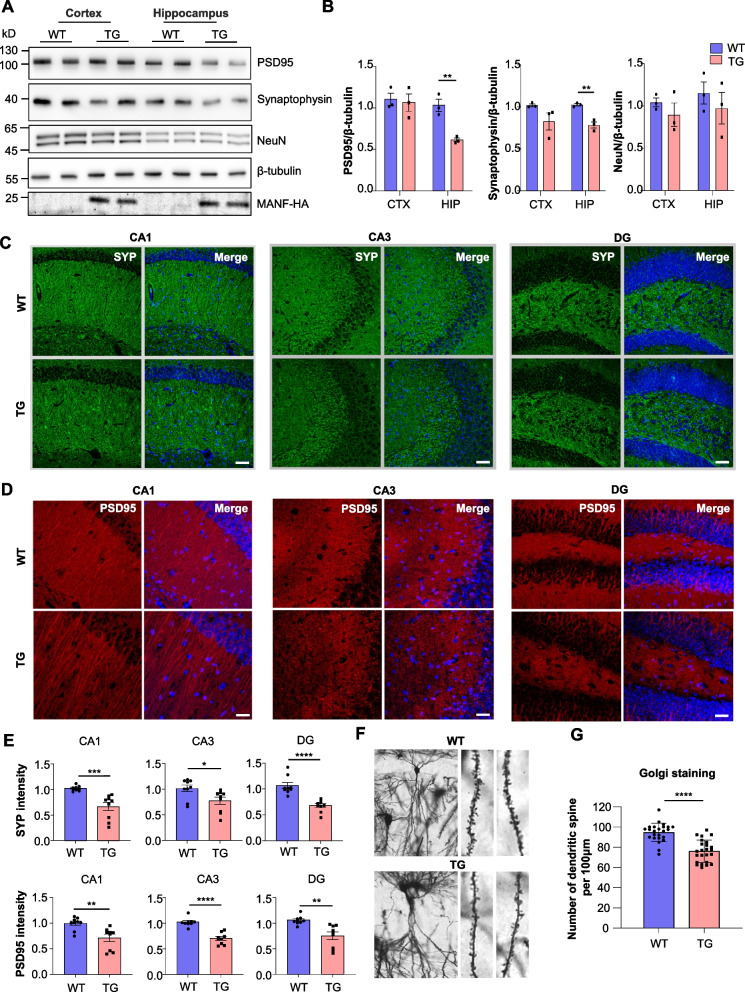
MANF transgenic mice exhibit synaptic defects. **A** Representative western blotting images of PSD95, synaptophysin and NeuN in the cortex and hippocampus of wild type (WT) and MANF transgenic (TG) mice at the age of 6 months. HA tag indicated mouse genotypes, and β-tubulin was used as a loading control. **B** Quantification of western blotting results in Fig. 3A (*n* = 3; two-tailed student t-test; PSD95, cortex, *P* = 0.7802, hippocampus, *P* = 0.0056; synaptophysin, cortex, *P* = 0.1318, hippocampus, *P* = 0.0040; NeuN, cortex, *P* = 0.3783, hippocampus, *P* = 0.4728). **C**, **D** Immunofluorescent staining images of synaptophysin (SYP) (**C**) and PSD95 (**D**) in the hippocampus of WT and MANF TG mice at the age of 6 months (scale bar: 50 μm). **E** Quantification of synaptophysin and PSD95 staining intensity (*n* = 9 from three mice; two-tailed student t-test; SYP, CA1, *P* = 0.0005, CA3, *P* = 0.0237, DG, *P* < 0.0001; PSD95, CA1, *P* = 0.0031, CA3, *P* < 0.0001, DG, *P* = 0.0015). **F** Representative Golgi staining images of neurons from the CA1 area of the hippocampus in WT and MANF TG mice. **G** Quantification of the number of synapses per 100 μm (n = 24 from three mice; two-tailed student t-test; *P* < 0.0001). Ns, non-significant, * *P* < 0.05, ** *P* < 0.01, *** *P* < 0.001, **** *P* < 0.001. Data are represented as mean ± SEM

The cognitive defects of the MANF transgenic mice led us to investigate synapse formation in the brain. We compared the expression of neuronal marker protein NeuN and synaptic marker proteins synaptophysin and PSD95 in WT and MANF transgenic mice at the age of 6 months. We found that while NeuN remained unchanged, both synaptophysin and PSD95 were significantly decreased in the hippocampus (Fig. 3A, B). By immunofluorescent staining, we confirmed that both synaptophysin and PSD95 were reduced in different hippocampal subregions including CA1, CA3, and dentate gyrus (DG) (Fig. 3C-E). We also performed synaptophysin and PSD95 co-staining to label synaptic boutons and found the density was significantly lower in MANF transgenic mice (Fig. S4A, B). Additionally, Golgi staining was used to directly visualize synapses in the hippocampus of MANF transgenic mice. The number of dendritic spines in the CA1 region of MANF transgenic mice was significantly lower than that of WT mice (Fig. 3F, G). Because microglia play an important role in synaptic pruning [47], we wonder if MANF affects microglia-mediated synapse phagocytosis. Neither the overall IBA1 staining intensity nor IBA1 and synaptophysin co-staining area was significantly changed in MANF transgenic mice (Fig. S4C, D). Together, these data indicate that MANF overexpression causes synapse loss in the hippocampus, which probably accounts for the learning and memory impairments of MANF transgenic mice.

### MANF overexpression in the hippocampus recapitulates the phenotypes in MANF transgenic mice

**Fig. 4:**
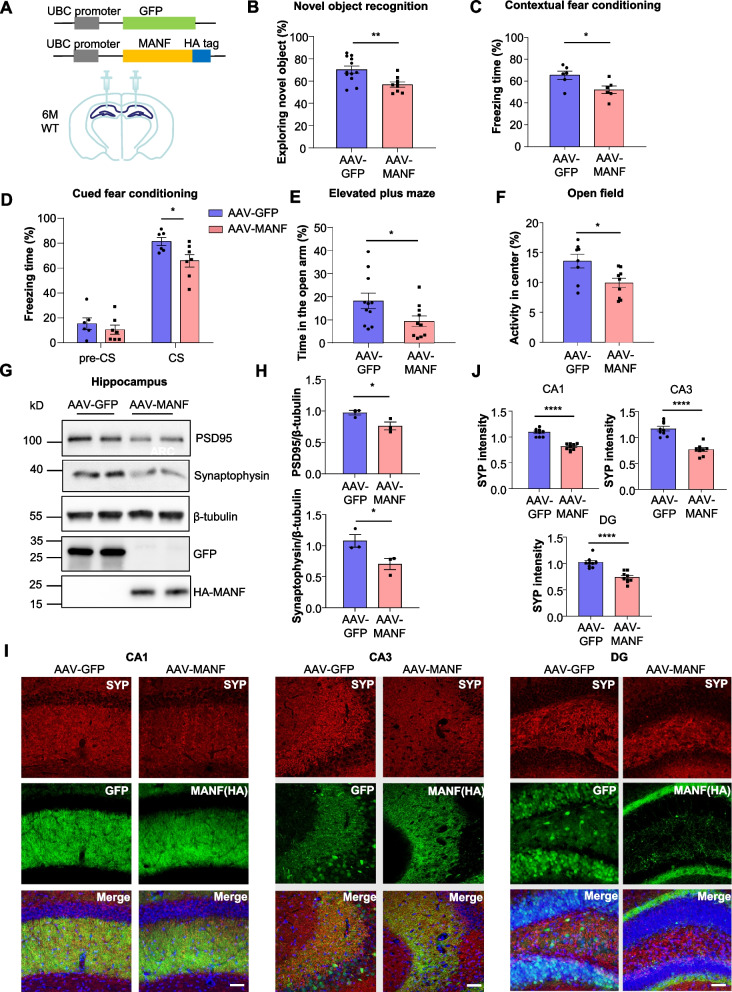
MANF overexpression in the hippocampus causes behavioral and synaptic deficits. **A** Schematic representation of AAV-GFP and AAV-MANF constructs. AAV-GFP or AAV-MANF were bilaterally injected into the hippocampus of WT mice at the age of 6 months. The analyses were performed one month after virus injection. **B** Novel object recognition test was performed on WT mice injected with AAV-GFP or AAV-MANF. The percentage of time spent exploring the novel object was calculated (*n* = 9–12; two-tailed student t-test; *P* = 0.0053). **C** Contextual fear conditioning test was on performed on WT mice injected with AAV-GFP or AAV-MANF. The percentage of freezing time in the same context was calculated (*n* = 6; two-tailed student t-test; *P* = 0.0273). **D** Cued fear conditioning test was on performed on WT mice injected with AAV-GFP or AAV-MANF. The percentage of freezing time before (pre-CS) and after conditioned stimulus (CS) was calculated (*n* = 6–7; two-tailed student t-test; *P* = 0.0332). **E** Elevated plus maze was performed on WT mice injected with AAV-GFP or AAV-MANF. The percentage of time spent in the open arm was calculated (*n* = 10–11; two-tailed student t-test; *P* = 0.0445). **F** Open field test was performed on WT mice injected with AAV-GFP or AAV-MANF. The percentage of time spent in the center was calculated (*n* = 8–9; two-tailed student t-test; *P* = 0.0171). **G** Representative western blotting images of synaptophysin and PSD95 in the hippocampus of WT mice injected with AAV-GFP or AAV-MANF. RFP and HA tag indicated the expression of AAVs, and β-tubulin was used as a loading control. **H** Quantification of western blotting results in Fig. 4G (*n* = 3; two-tailed student t-test; PSD95, *P* = 0.0432; synaptophysin, *P* = 0.0489). **I** Immunofluorescent staining images of synaptophysin (SYP) in the hippocampus of WT mice injected with AAV-GFP or AAV-MANF. GFP fluorescence and HA tag were used to indicate the expression of AAV-GFP and AAV-MANF respectively (scale bar:50 μm). **J** Quantification of synaptophysin staining intensity in the CA1, CA3, and dentate gyrus (DG) areas (*n* = 9 from three mice; two-tailed student t-test; CA1, *P* < 0.0001; CA3, *P* < 0.0001; DG, *P* < 0.0001). * *P* < 0.05, ** *P* < 0.01, *** *P* < 0.001, **** *P* < 0.001. Data are represented as mean ± SEM

The prion promoter drives MANF overexpression throughout the brain in the transgenic mouse model. To determine whether MANF overexpression in the hippocampus alone leads to similar phenotypes, we generated adeno-associated virus expressing MANF with an HA tag (AAV-MANF) and injected with virus into both sides of the hippocampus in 6-month-old WT mice (Fig. 4A). One month after viral injection, we found that the mice injected with AAV-MANF performed significantly worse in novel object recognition and fear conditioning tests, compared with the control mice injected with AAV-GFP (Fig. 4B-D). The mice injected with AAV-MANF also displayed heightened anxiety in the elevated plus maze and open field tests (Fig. 4E, F).

After the behavioral tests, we sacrificed the mice and collected the brain tissues for neuropathological analyses. Some of the brains were used for western blotting and others for immunofluorescent staining. Compared with AAV-GFP injected mice, AAV-MANF injected mice had significantly decreased expression of synaptophysin and PSD95 in the hippocampus (Fig. 4G, H). The same results were demonstrated by immunofluorescent staining of different hippocampal regions in the AAV-MANF injected mice (Fig. 4I, J, S5A, B). Together, these results suggest that overexpression of MANF specifically in the hippocampus can recapitulate the behavioral defects and synapse loss in the MANF transgenic mice.

### MANF interacts with the RNA-binding protein ELAVL2

**Fig. 5:**
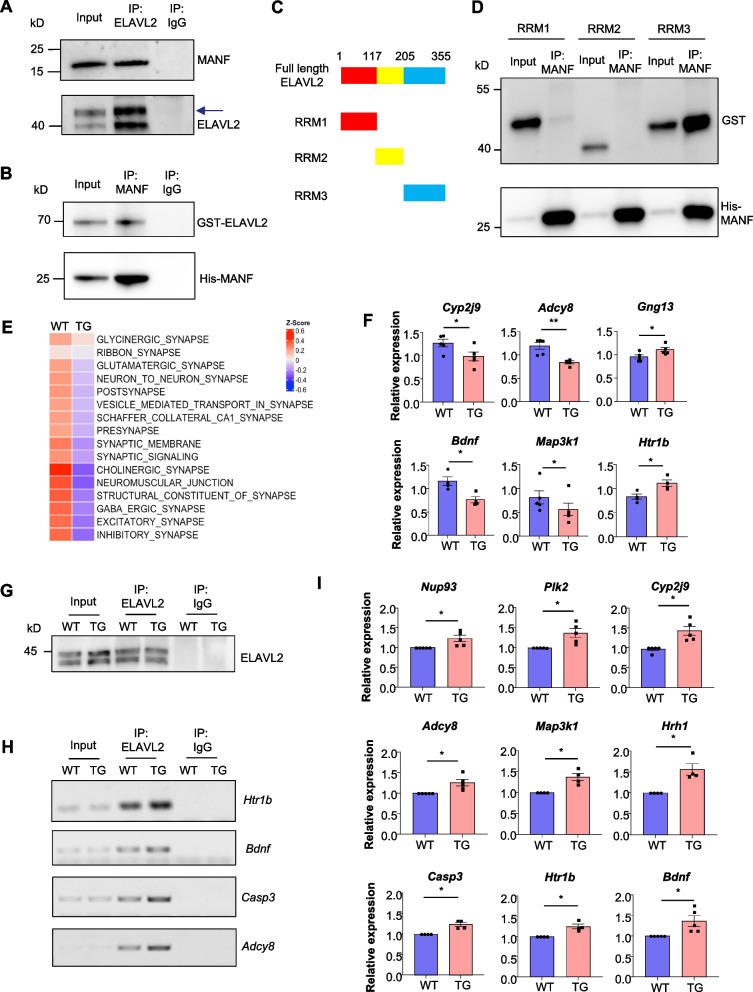
MANF interacts with ELAVL2 and mediates its RNA-binding properties. **A** Co-immunoprecipitation assay was performed using WT mouse brain lysate and ELAVL2 antibody. Representative western blotting images showed that MANF was detected in the pull-down lysate. Rabbit IgG was used as a negative control. The lower band represents ELAVL2, and the upper band (indicated by an arrow) represents its homolog ELAVL4. **B** In vitro binding assay was performed using recombinant GST-ELAVL2 and His-MANF proteins. Representative western blotting images showed that GST-ELAVL2 was detected in the MANF antibody pull-down lysate. Rabbit IgG was used as a negative control. **C** Schematic representation of GST tagged full length ELAVL2 and ELAVL2 fragments (RRM1, RRM2 and RRM3). **D** In vitro binding assay was performed using recombinant GST tagged ELAVL2 fragments (RRM1, RRM2 and RRM3) and His-MANF proteins. Representative western blotting images showed that the fragment RRM3 was detected in the MANF antibody pull-down lysate. **E** Heatmap showing the relative transcripts enrichment of the selected GO pathways related to synapse. **F** Quantitative real-time PCR analysis of selected genes using mRNAs extracted from the hippocampus of WT and TG mice (*n* = 4–5; two-tailed student t-test; *Cyp2j9*, *P* = 0.0487; *Adcy8*, *P* = 0.0055; *Gng13*, *P* = 0.0388; *Bdnf*, *P* = 0.0134; *Map3k1*, *P* = 0.0492; *Htr1b*, *P* = 0.0168). **G** RNA immunoprecipitation (RIP) assay was performed using the hippocampus of WT and TG mice. Representative western blotting images showed that ELAVL2 was pulled down. Rabbit IgG was used as a negative control. **H** Reverse transcriptase PCR analysis of selected genes using mRNAs purified from the RIP assays. **I** Quantitative real-time PCR analysis of selected genes using mRNAs purified from the RIP assays (*n* = 4–5; two-tailed student t-test; *Nup93*, *P* = 0.0416; *Plk2*, *P* = 0.0287; *Cyp2j9*, *P* = 0.0314; *Adcy8, P* = 0.0323; *Map3k1*, *P* = 0.0229; *Hrh1*,* P* = 0.0257; *Casp3*, *P* = 0.0155; *Htr1b*, *P* = 0.0222; *Bdnf*, *P* = 0.0447). Ns, non-significant, * *P* < 0.05, ** *P* < 0.01. Data are represented as mean ± SEM

Next, we aim to study the mechanism of MANF in regulating synaptic functions. We previously identified MANF interactome by affinity purification and mass spectrometry [27]. Through the list of MANF interacting proteins, we focused on ELAVL2, which is an RNA-binding protein that controls mRNA stability. ELAVL2 plays important roles in regulating synaptic functions and has been implicated in AD [48–50]. To verify the interaction between ELAVL2 and MANF, we performed a co-immunoprecipitation assay using the brain homogenates of WT mice. We found that endogenous MANF was co-immunoprecipitated with ELAVL2 (Fig. 5A). We also purified recombinant GST-tagged ELAVL2 protein (GST-ELAVL2) and His tagged MANF protein (His-MANF) and confirmed the direct binding between ELAVL2 and MANF (Fig. 5B). The full-length ELAVL2 protein is composed of three RNA recognition motifs (RRMs) [51, 52]. To identify which motif within ELAVL2 directly interacts with MANF, we generated truncated ELAVL2 proteins corresponding to RRM1, RRM2, and RRM3 respectively (Fig. 5C). By incubating each of the ELAVL2 fragments with His-MANF and pulling down His-MANF, we found that His-MANF preferentially interacted with RRM3 (Fig. 5D), suggesting that the interaction between MANF and ELAVL2 is mediated by the RRM3 domain.

We examined the expression of ELAVL2 in different brain regions of adult WT mice and found that the expression of ELAVL2 was high in the cerebellum but low in the hippocampus (Fig. S6A, B). Therefore, the function of ELAVL2 in the hippocampus could be particularly sensitive to fluctuations in MANF expression. We also compared the expression of ELAVL2 between WT and MANF transgenic mice but found no significant differences in the cortex and hippocampus (Fig. S6C, D). In addition, we performed subcellular fractionation using the brain homogenates of WT and MANF transgenic mice. Both ELAVL2 and MANF were predominantly localized in the cytoplasm, and MANF overexpression did not alter the cellular localization of ELALV2 (Fig. S6E, F).

### MANF overexpression affects the stability of RNA transcripts mediated by ELAVL2

To investigate whether MANF overexpression alters RNA transcripts in the hippocampus, we performed RNA sequencing (RNAseq) analysis using 6-month-old WT and MANF transgenic mice. The transcriptome of WT and MANF transgenic mice was not substantially different, which agrees with the lack of obvious neuronal loss in MANF transgenic mice. Employing an unadjusted *P* value of < 0.05 as the threshold for statistical significance, we identified 172 differentially expressed genes (DEGs) in the hippocampus of MANF transgenic mice in comparison to the WT mice, including 66 up-regulated genes and 106 down-regulated genes (Fig. S7A). GO analysis revealed that “Translation at synapse” is among the pathways that are most significantly affected (Fig. S7B). We also found that a significant portion of the DEGs have functions closely related to neurons and synapses (Fig. S7C). In addition, the overall transcription level of genes involved in the synapse-related GO pathways was lower in MANF transgenic mice, compared with WT mice (Fig. 5E). To validate the RNAseq analyses, we selected a few DEGs (*Adcy8*, *Bdnf*, *Cyp2j9*, *Gng13*, *Htr1b*, and *Map3k1*) that have been linked to neuronal and synaptic functions in previous studies [53–58] for quantitative real-time PCR. We verified that the expression of these genes was significantly changed in the hippocampus of MANF transgenic mice (Fig. 5F), which agrees with the RNAseq results.

The RRM3 of ELAVL2 binds to the polyA tail of selected transcripts and mediates their stability [51]. To verify if ELAVL2 binds to the mRNAs of the identified DEGs, we performed an RNA immunoprecipitation (RIP) assay using the hippocampus of WT and MANF transgenic mice. We immunoprecipitated ELAVL2 by its antibody (Fig. 5G), and purified mRNAs from the immunoprecipitants for reverse transcriptase PCR. The results demonstrated that more mRNAs were co-purified with ELAVL2 from the hippocampus of MANF transgenic mice (Fig. 5H), suggesting that MANF overexpression enhances the binding of ELAVL2 to certain mRNAs. We examined more DEGs using the mRNAs from the RIP assay and the quantitative real-time PCR results showed that significantly more transcripts were co-immunoprecipitated with ELAVL2 in the MANF transgenic mice, compared with WT mice (Fig. 5I). Together, these results indicate that the stability of certain ELAVL2-bond transcripts is affected in the hippocampus of MANF transgenic mice, leading to synaptic dysfunctions.

### MANF overexpression exacerbates pathological phenotypes of 5xFAD mice

**Fig. 6:**
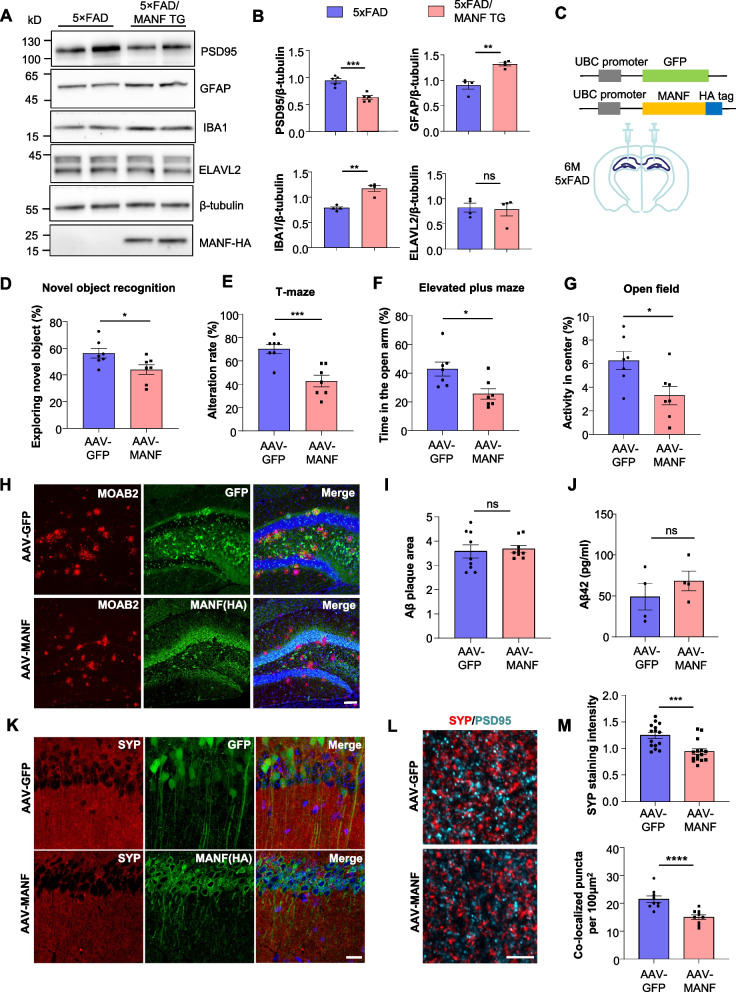
Overexpressing MANF exacerbates behavioral deficits and synaptic pathology in 5xFAD mice. **A** Representative western blotting images of PSD95, GFAP, IBA1, and ELAVL2 in the hippocampus of 5xFAD and 5xFAD/MANF TG mice at the age of 9 months. HA tag indicated mouse genotypes, and β-tubulin was used as a loading control. **B** Quantification of western blotting results in Fig. 6B (*n* = 4–5; two-tailed student t-test; PSD95, *P* = 0.0005; GFAP, *P* = 0.0021; IBA1, *P* = 0.0011; ELAVL2, *P* = 0.8161). **C** AAV-GFP or AAV-MANF was injected into both sides of the hippocampus in 5xFAD mice at the age of 6 months. The analyses were performed one month after virus injection. **D** Novel object recognition test was performed on 5xFAD mice injected with AAV-GFP or AAV-MANF. The percentage of time spent exploring the novel object was calculated (*n* = 7; two-tailed student t-test; *P* = 0.0349). **E** T-maze test was performed on 5xFAD mice injected with AAV-GFP or AAV-MANF. The alteration rate was calculated (*n* = 7; two-tailed student t-test; *P* = 0.0009). **F** Elevated plus maze was performed on 5xFAD mice injected with AAV-GFP or AAV-MANF. The percentage of time spent in the open arm was calculated (*n* = 7; two-tailed student t-test; *P* = 0.0148). **G** Open field test was performed on 5xFAD mice injected with AAV-GFP or AAV-MANF. The percentage of time spent in the center was calculated (*n* = 7; two-tailed student t-test; *P* = 0.0192). **H** Immunofluorescent staining images of Aβ plaques using the MOAB2 antibody in the hippocampus of 5xFAD mice injected with AAV-GFP or AAV-MANF (scale bar: 100 μm). **I** Quantification of Aβ plaque area in Fig. 6H (*n* = 9 from three mice, two-tailed student t-test; *P* = 0.7304). **J** ELISA was performed to assess the concentration of Aβ42 in the hippocampus of 5xFAD mice injected with AAV-GFP or AAV-MANF (*n* = 4; two-tailed student t-test; *P* = 0.3655). (**I**) Immunofluorescent staining images of synaptophysin in the hippocampus of 5xFAD mice injected with AAV-GFP or AAV-MANF (scale bar: 20 μm). (**L**) Double-immunofluorescent staining images of synaptophysin and PSD95 in the hippocampus of 5xFAD mice injected with AAV-GFP or AAV-MANF (scale bar: 5 μm). **M** Quantification of synaptophysin staining intensity (*n* = 15 from three mice; two-tailed student t-test; *P* = 0.0006) and synaptophysin/PSD95 co-localized puncta (*n* = 9 from three mice; two-tailed student t-test; *P* < 0.0001) in the hippocampus of 5xFAD mice injected with AAV-GFP or AAV-MANF. Ns, non-significant, * *P* < 0.05, ** *P* < 0.01, *** *P* < 0.001, **** *P* < 0.0001. Data are represented as mean ± SEM

We wonder if increasing MANF expression can accelerate disease progression in 5xFAD mice. To that end, we crossed the MANF transgenic mice with 5xFAD mice. In the 9-month-old mice that carry both transgenes (5xFAD/MANF TG), we found a significant reduction of PSD95 and a significant increase of GFAP and IBA1 compared with age-matched 5xFAD mice, although the expression of ELAVL2 was unchanged (Fig. 6A, B).

We also performed stereotaxic injection of AAV-MANF in the hippocampus of 6-month-old 5xFAD mice and examined their behaviors one month after surgery (Fig. 6C). AAV-MANF was primarily expressed in neurons but not in astrocytes (Fig. S8A). In novel object recognition and T-maze tests, the 5xFAD mice injected with AAV-MANF performed significantly worse than the 5xFAD mice injected with AAV-GFP (Fig. 6D, E), indicating cognitive impairments. The 5xFAD mice injected with AAV-MANF also displayed heightened anxiety, as manifested by elevated plus maze and open field tests (Fig. 6F, G). After the behavioral analyses, we collected the brain tissues and examined Aβ burden via immunofluorescent staining with the MOAB2 antibody and Aβ42 ELISA. Neither Aβ plaque area nor Aβ42 concentration was significantly changed in 5xFAD mice injected with AAV-MANF (Fig. 6H-J), suggesting that MANF overexpression does not alter Aβ burden in the brain. Nonetheless, immunofluorescent staining with the synaptophysin and PSD95 antibodies revealed that MANF overexpression led to a more severe loss of synapses in the hippocampus of 5xFAD mice (Fig. 6K-M). We also found a significant increase in GFAP and IBA1 staining intensity and IBA1/synaptophysin co-staining area in the AAV-MANF infected brain (Fig. S8B-E), indicating glial activation and enhanced synapse phagocytosis by microglia.

### Reducing MANF expression attenuates pathological phenotypes of 5xFAD mice

**Fig. 7:**
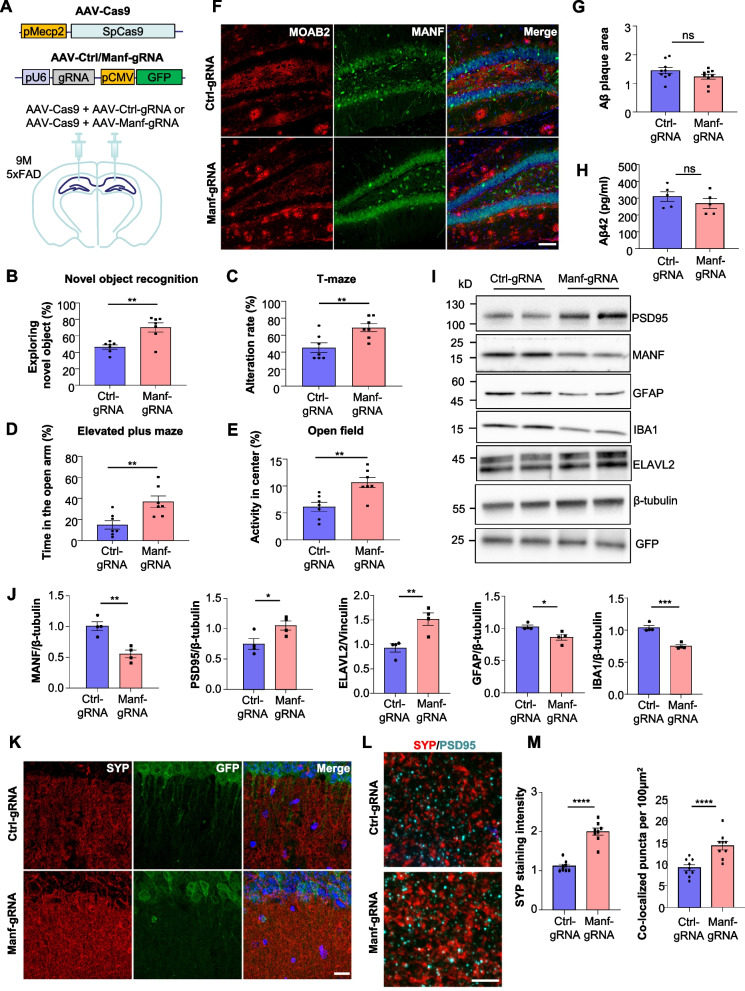
Reducing MANF ameliorates behavioral deficits and synapse loss in 5xFAD mice. **A** Schematic representation of AAV-Cas9, AAV-Ctrl-gRNA and AAV-Manf-gRNA constructs. AAV-Cas9/AAV-Manf-gRNA or AAV-Cas9/AAV-Ctrl-gRNA was injected into both sides of the hippocampus in 5xFAD mice at the age of 9 months. The analyses were performed three months after virus injection. **B** Novel object recognition test was performed on 5xFAD mice injected with AAV-Cas9/AAV-Manf-gRNA or AAV-Cas9/AAV-Ctrl-gRNA. The percentage of time spent exploring the novel object was calculated (*n* = 7; two-tailed student t-test; *P* = 0.0022). **C** T-maze test was performed on 5xFAD mice injected with AAV-Cas9/AAV-Manf-gRNA or AAV-Cas9/AAV-Ctrl-gRNA. The alteration rate was calculated (*n* = 7; two-tailed student t-test; *P* = 0.0081). **D** Elevated plus maze was performed on 5xFAD mice injected with AAV-Cas9/AAV-Manf-gRNA or AAV-Cas9/AAV-Ctrl-gRNA. The percentage of time spent in the open arm was calculated (*n* = 7; two-tailed student t-test; *P* = 0.0061). **E** Open field test was performed on 5xFAD mice injected with AAV-Cas9/AAV-Manf-gRNA or AAV-Cas9/AAV-Ctrl-gRNA. The percentage of time spent in the center was calculated (*n* = 7; two-tailed student t-test; *P* = 0.0035). **F** Immunofluorescent staining images of Aβ plaques using the MOAB2 antibody in the hippocampus of 5xFAD mice injected with AAV-Cas9/AAV-Manf-gRNA or AAV-Cas9/AAV-Ctrl-gRNA (scale bar: 100 μm). **G** Quantification of Aβ plaque area in Fig. 7F (*n* = 9 from three mice; two-tailed student t-test; *P* = 0.1470). **H** ELISA was performed to assess the concentration of Aβ42 in the hippocampus of 5xFAD mice injected with AAV-Cas9/AAV-Manf-gRNA or AAV-Cas9/AAV-Ctrl-gRNA (*n* = 5; two-tailed student t-test; *P* = 0.3368). **I** Representative western blotting images of PSD95, MANF, GFAP, IBA1 and ELAVL2 in the hippocampus of 5xFAD mice injected with AAV-Cas9/AAV-Ctrl-gRNA or AAV-Cas9/AAV-Manf-gRNA. GFP indicated the expression of AAVs. β-tubulin was used as a loading control. **J** Quantification of western blotting results in Fig. 7I (*n* = 4; two-tailed student t-test; MANF, *P* = 0.0033; PSD95, *P* = 0.0389; ELAVL2, *P* = 0.0081; GFAP, *P* = 0.0187; IBA1, *P* = 0.0004). **K** Immunofluorescent staining images of synaptophysin in the hippocampus of 5xFAD mice injected with AAV-Cas9/AAV-Manf-gRNA or AAV-Cas9/AAV-Ctrl-gRNA (scale bar: 20 μm). **L** Double-immunofluorescent staining images of synaptophysin and PSD95 in the hippocampus of 5xFAD mice injected with AAV-Cas9/AAV-Manf-gRNA or AAV-Cas9/AAV-Ctrl-gRNA (scale bar: 5 μm). **M** Quantification of synaptophysin staining intensity and synaptophysin/PSD95 co-localized puncta in the hippocampus of 5xFAD mice injected with AAV-Cas9/AAV-Manf-gRNA or AAV-Cas9/AAV-Ctrl-gRNA (*n* = 9 from three mice; two-tailed student t-test; synaptophysin, *P* < 0.0001; synaptophysin/PSD95, *P* < 0.0001). Ns, non-significant, * *P* < 0.05, ** *P* < 0.01, *** *P* < 0.001, **** *P* < 0.0001. Data are represented as mean ± SEM

We also adopted the opposite approach, in which CRISPR/Cas9 viruses were delivered to the hippocampus of 9-month-old 5xFAD mice via stereotaxic injection to knock down endogenous MANF expression (Fig. 7A). Three months after viral injection, the 5xFAD mice injected with AAV-Cas9/AAV-Manf-gRNA had significantly improved performances in cognition tests including novel object recognition and T-maze, compared with those injected with AAV-Cas9/AAV-Ctrl-gRNA (Fig. 7B, C). MANF reduction also alleviated anxiety-related behaviors in 5xFAD mice as demonstrated by the elevated plus maze and open field tests (Fig. 7D, E). Via immunofluorescent staining and ELISA, we found that Aβ burden in these mice was unaffected (Fig. 7F-H). The reduction of MANF was accompanied by a significant increase of PSD95 and ELAVL2, and a substantial decrease of GFAP and IBA1, indicating alleviated synapse loss and neuronal injury (Fig. 7I, J).

We also performed immunofluorescent staining using the hippocampal slices. The reduction of MANF expression was confirmed in the cells expressing GFP fluorescence (Fig. S9A). Importantly, in the hippocampal areas infected with AAV-Cas9/AAV-Manf-gRNA, the staining intensity of synaptophysin and PSD95 was significantly increased (Fig. 7K-M), suggesting that MANF knockdown ameliorates synaptic pathology in 5xFAD mice. MANF reduction also attenuated astrocyte and microglia activation and microglia-mediated synapse phagocytosis (Fig. S9D, E).

## Discussion

Synapse loss is an early pathological feature of AD and is the strongest correlate to the cognitive symptoms of AD patients [1–3]. ER stress triggered by altered proteostasis also happens before the emergence of clinical symptoms in AD patients [8, 9]. ER stress has been linked to the synaptic pathology in AD, as the ER stress sensor XBP1 mediates hippocampal synaptic plasticity in AD mice [59, 60]. Nonetheless, mechanistic insights connecting ER stress and synaptic dysfunction remain lacking. In the present study, we identified the ER stress inducible protein MANF as a negative regulator of synaptic function during AD pathogenesis. Strong evidence in support of our claim includes: first, the increased expression of MANF correlated with synapse loss in the hippocampus of AD mice; second, the ectopic expression of MANF in the hippocampus led to synapse loss and behavioral deficits that are reminiscent of AD mice; third, increasing or decreasing MANF expression exacerbated or ameliorated the synaptic pathology in AD mice.

MANF is considered a protective molecule in several neurological diseases, including PD, ischemic stroke, and retinal degeneration [13, 15, 16]. The finding that MANF contributes to AD pathogenesis may seem inconsistent at first glance. Nonetheless, it is worth noting that MANF was administered as a recombinant protein in these studies. The neuroprotective effects of the recombinant MANF protein could be due to pro-survival signaling pathways such as AKT, ERK or PKC [28, 61], although the transmembrane receptor through which MANF exerts its functions remains unknown. Intracellularly, MANF functions as an ER stress inducible protein. There are extensive evidence showing that the pancreatic β cells deficient in MANF are prone to apoptosis due to elevated ER stress [62, 63], whereas brain knockout of MANF does not cause obvious neuronal loss or neurological phenotypes [64]. Therefore, the outcome of altered MANF expression in the ER appears to be cell-type specific.

One previous study reported elevated expression of MANF in the brains of both pre-symptomatic and symptomatic AD patients [25], but the consequence of the early rise in MANF protein remains unexplored. In cultured cells, MANF overexpression protects against Aβ42 induced cell death via inhibiting ER stress [26]. However, this observation is based on an acute in vitro model. ER stress initially triggers adaptive UPR, which helps to maintain ER homeostasis, but excessive and prolonged ER stress cause maladaptive UPR that has detrimental effects. The main difference between adaptive and maladaptive UPR is the level and duration of ER stress, as they share similar signaling pathways [65, 66]. Our findings that sustained upregulation of MANF leads to synaptic defects are consistent with such a paradigm.

We identified ELAVL2 as a mechanistic link between MANF overexpression and synaptic defects. The ELAVL family of RNA-binding proteins plays important roles in mediating the stability of RNA transcripts. Emerging evidence indicates that the dysfunctions of ELAVLs contribute to neurodevelopmental and neurodegenerative diseases. For example, ELAVL2 has been linked to learning and memory formation, autism spectrum disorder and schizophrenia [48, 49, 67]; ELAVL3 has been linked to amyotrophic lateral sclerosis (ALS) and cerebellar ataxia [68, 69]; ELAVL4 has been linked to AD and PD [50, 70, 71]. Through in vitro binding assay, we confirmed the direct interaction between ELAVL2 and MANF via the RRM3 domain. Nonetheless, given the high sequence homology between ELAVL isoforms (79% between ELAVL2 and ELAVL3, 87% between ELAVL2 and ELAVL4) and that other ELAVL isoforms are also co-immunoprecipitated with MANF, we cannot rule out the possibility that MANF broadly affects the functions of multiple ELAVLs. ELAVL2 is known to regulate the transcription of synaptic genes [48]. Through RNAseq and RIP assay, we found that MANF overexpression enhanced the RNA-binding capacity of ELAVL2. Interestingly, the augmented binding does not consistently translate into more RNA transcripts, indicating a gene-specific influence of ELAVL2 on RNA transcript stability. We also noted that knocking down MANF increased the expression of ELVAL2 in 5xFAD mice, suggesting a multifaceted impact of MANF on ELAVL2, which may encompass both protein binding and expression. In addition, the hippocampus has a relatively low level of ELAVL2, whereas the cerebellum has the highest level of ELAVL2 among different brain regions examined. Accordingly, MANF overexpression preferentially affects neuronal functions in the hippocampus but protects Purkinje neurons in the presence of misfolded polyglutamine proteins [28]. These results suggest that the brain region-specific effects of MANF overexpression could be caused by variations in ELAVL2 expression.

MANF is actively being pursued as a therapeutic target for treating neurological diseases such as PD. Our study has important implications for the development of MANF-based therapies. First, considering the complex functions of MANF within the cell, the ectopic expression of MANF via AAVs could potentially cause negative effects. Therefore, the extracellular delivery of recombinant MANF protein should be a safer and more effective approach. Our previous finding that AAV-mediated overexpression of MANF in the hypothalamus leads to insulin resistance and hyperphagia agrees with this conclusion [27]. Second, our results showed that MANF knockdown significantly attenuates synapse loss in 5xFAD mice. As removal of MANF in the brain does not cause obvious defects in mice [64], the MANF-lowering method could be a potential therapy for treating AD. Reducing MANF expression via genetic approaches in the context of AD is warranted to further validate this point.

## Conclusions

In summary, we found that the increased expression of MANF correlates with synapse loss in the hippocampus of AD mice. MANF overexpression in mouse hippocampus causes learning and memory deficits and synaptic dysfunctions. Mechanistically, MANF interacts with ELAVL2 and mediates its binding to transcripts that are involved in synaptic functions. Increasing or decreasing MANF expression in the hippocampus exacerbates or ameliorates disease phenotypes in AD mice, respectively. Together, these findings suggest that ER-stress induced MANF expression contributes to synapse loss in AD. Reducing MANF expression could be employed as a potential therapeutic approach in AD treatment.

## Supplementary Information

Supplementary Material 1.

Supplementary Material 2.

## Data Availability

All data are available in the main text or the supplementary materials. The RNAseq data has been uploaded to the BioProject database under the accession number PRJNA1032076. Research materials are available from the corresponding author on reasonable request.

## Abbreviations

ER: Endoplasmic reticulum
AD: Alzheimer's disease
MANF: Mesencephalic astrocyte-derived neurotrophic factor
WT: Wild type
ELAVL2: ELAV like RNA-binding protein 2
UPR: Unfolded protein response
PD: Parkinson’s disease
GRP78: Glucose-regulated protein 78
DG: Dentate gyrus
RRM: RNA recognition motif
DEG: Differentially expressed gene
RIP: RNA immunoprecipitation
ALS: Amyotrophic lateral sclerosis
