# Supplementary material for: Increased expression of mesencephalic astrocyte-derived neurotrophic factor (MANF) contributes to synapse loss in Alzheimer’s disease
Source: Mol Neurodegener. 2024 Oct 18;19:75. doi: 10.1186/s13024-024-00771-3 (PMC11490049; doi:10.1186/s13024-024-00771-3)
Supplement: Supplementary file 1 — Supplementary Material 1. [file 13024_2024_771_MOESM1_ESM.pdf]

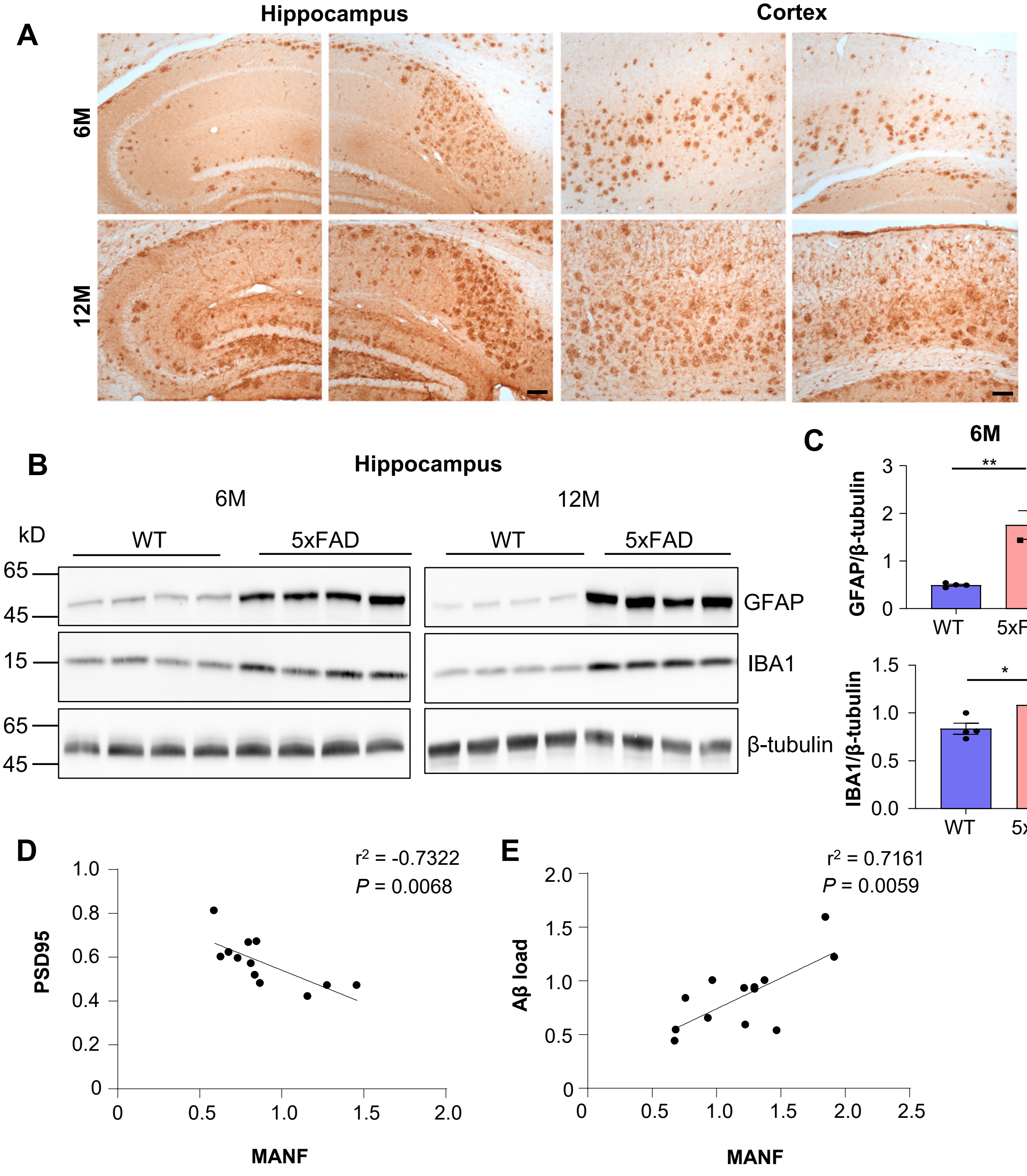

Figure S1. Characterization of neuropathology in the brain of AD mice (related to Figure 1). (A) Immunohistochemistry images showing Aβ plaques in the brain of 5xFAD mice at the age of 6 and 12 months (scale bar: 100 μm). **(B)** Representative western blotting images of GFAP and IBA1 expression in the hippocampus of WT and 5xFAD mice at the age of 6 and 12 months. **(C)** Quantification of western blotting results in Fig. S1B (n = 4; two-tailed student t-test; 6M, GFAP, $P = 0.0056$, IBA1, $P = 0.0376$; 12M, GFAP, $P < 0.0001$; IBA1, $P < 0.0001$). **(D)** Linear regression analysis of the correlation between MANF and PSD95 expression detected by western blotting. **(E)** Linear regression analysis of between MANF expression detected by western blotting and Aβ load detected by ELISA. * $P < 0.05$, ** $P < 0.01$, **** $P < 0.0001$. Data are represented as mean ± SEM.

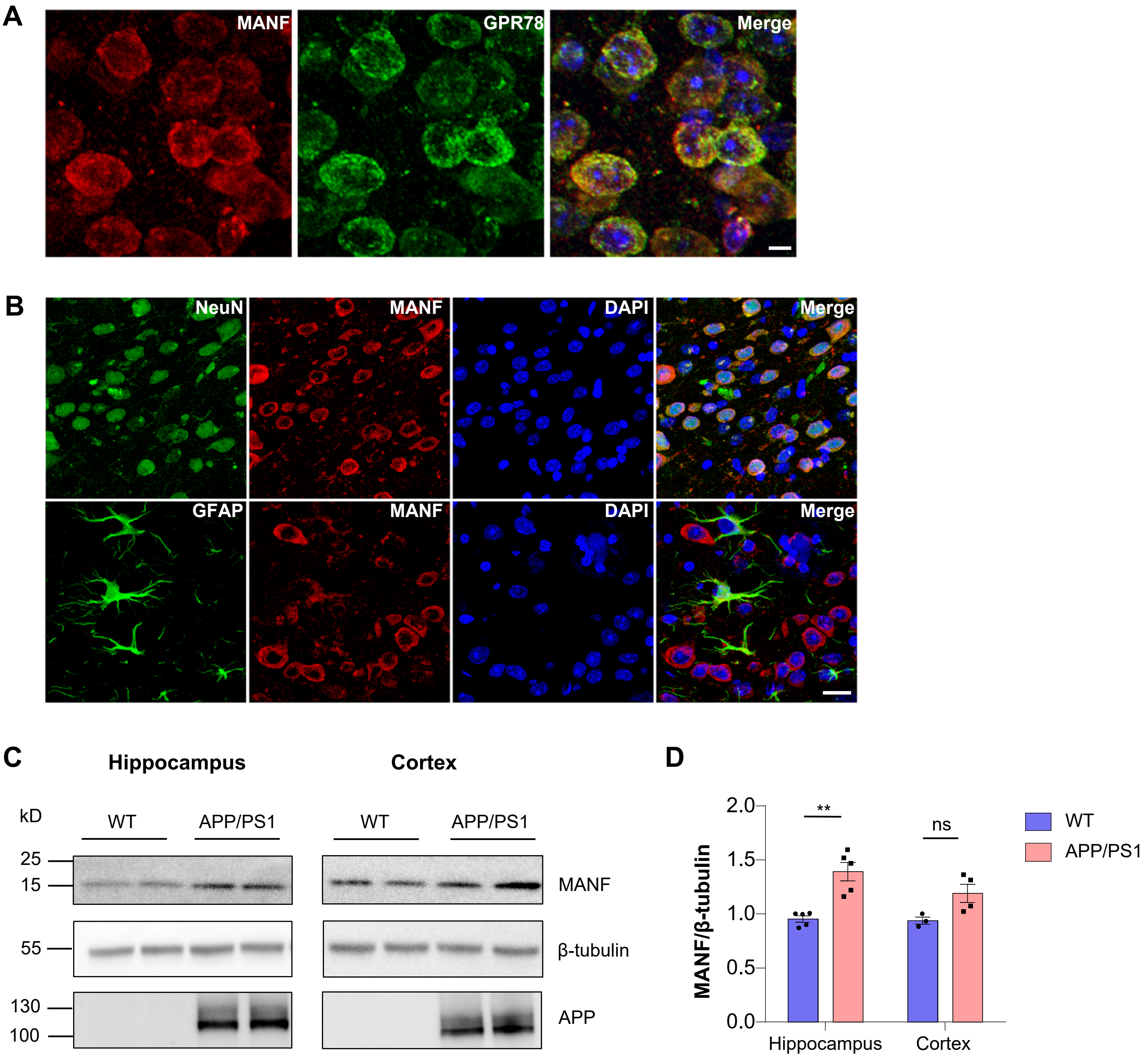

Figure S2. Characterization of MANF expression in the brain of AD mice (related to Figure 1). (A) Double-immunofluorescent staining images of MANF and GPR78 in WT mouse brain (scale bar: 10 μm). **(B)** Double-immunofluorescent staining images of MANF and NeuN, or MANF and GFAP in the brain of 5xFAD mice (scale bar: 20 μm). **(C)** Representative western blotting images of MANF expression in the hippocampus and cortex of APP/PS1 mice at the age of 12 months. APP was used to indicate mouse genotypes. **(D)** Quantification of western blotting results in Fig. S2C (n = 3-5; two-tailed student t-test; hippocampus, $P = 0.0012$; cortex, $P = 0.0598$). Ns, non-significant, ** $P < 0.01$. Data are represented as mean ± SEM.

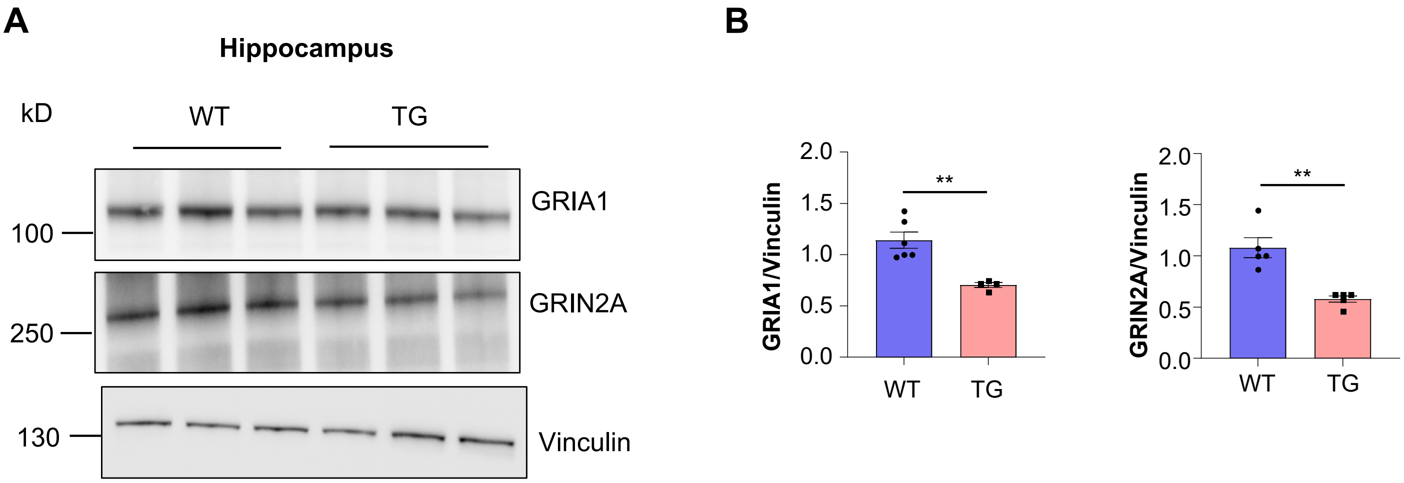

Figure S3. The expression of glutamate receptors are decreased in the hippocampus of MANF transgenic mice (related to Figure 2). (A) Representative western blotting images of GRIA1 and GRIN2A in the hippocampus of wild-type (WT) and MANF transgenic (TG) mice at the age of 6 months. Vinculin was used as a loading control. **(B)** Quantification of western blotting results in Fig. S3A (n = 4-6; two-tailed student t-test; GRIA1, $P = 0.0023$; GRIN2A, $P = 0.0013$). ** $P < 0.01$. Data are represented as mean ± SEM.

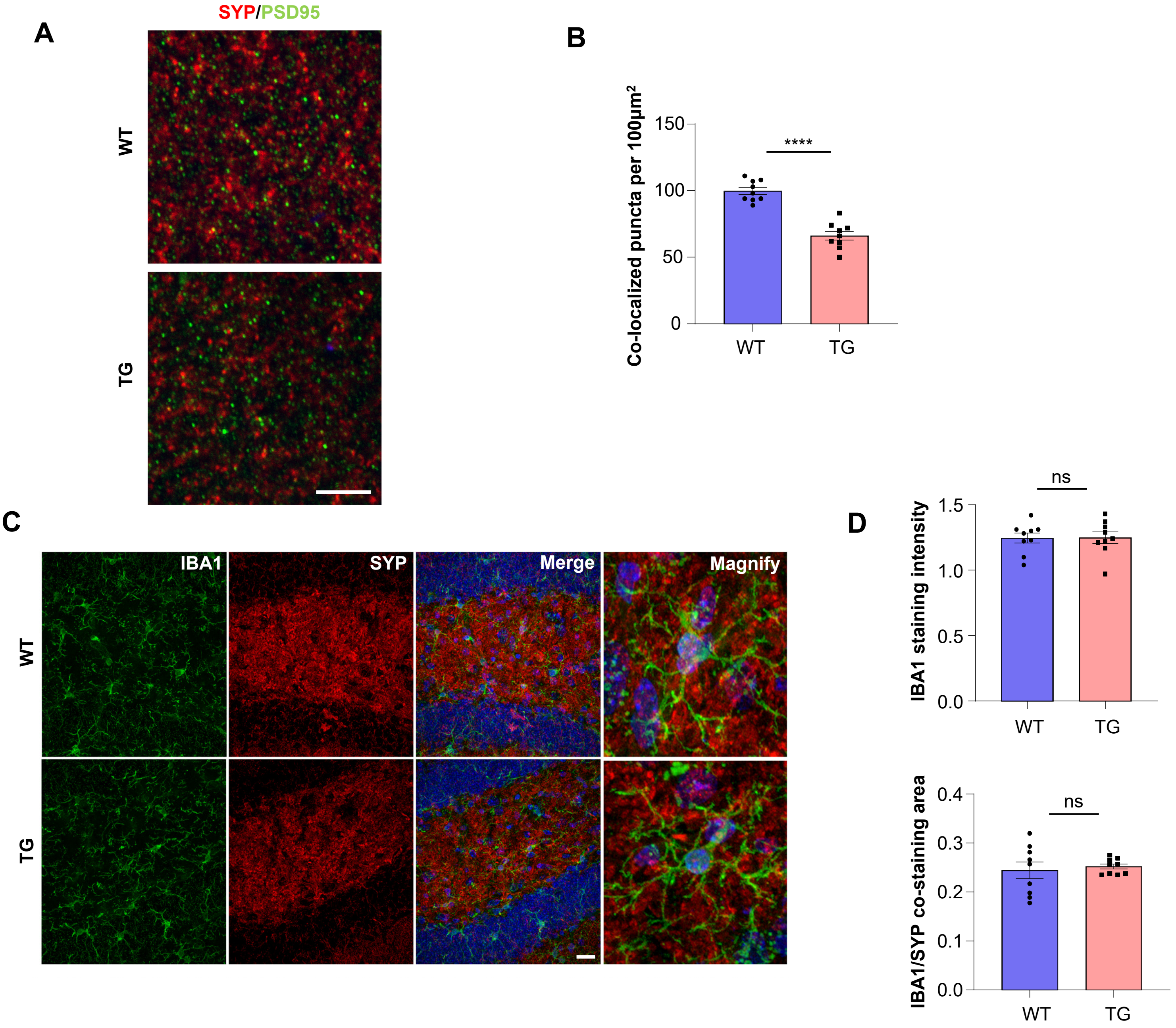

Figure S4. Further characterization of synspse loss in the hippocampus of MANF transgenic mice (related to Figure 3). (A) Double-immunofluorescent staining images of synaptophysin (SYP) and PSD95 in the hippocampus of WT and MANF transgenic (TG) mice (scale bar: 5 μm). **(B)** Quantification of SYP/PSD95 co-localized puncta (n = 9 from three mice; two-tailed student t-test; $P < 0.0001$). **(C)** Double-immunofluorescent staining images of IBA1 and SYP in the hippocampus of WT and MANF TG mice (scale bar: 50 μm). **(D)** Quantification of IBA1 staining intensity and IBA1/SYP co-staining area (n = 9 from three mice; two-tailed student t-test; IBA1, $P = 0.9703$; IBA1/SYP, $P = 0.6698$). Ns, non-significant, ** $P < 0.01$. Data are represented as mean ± SEM.

Figure S5

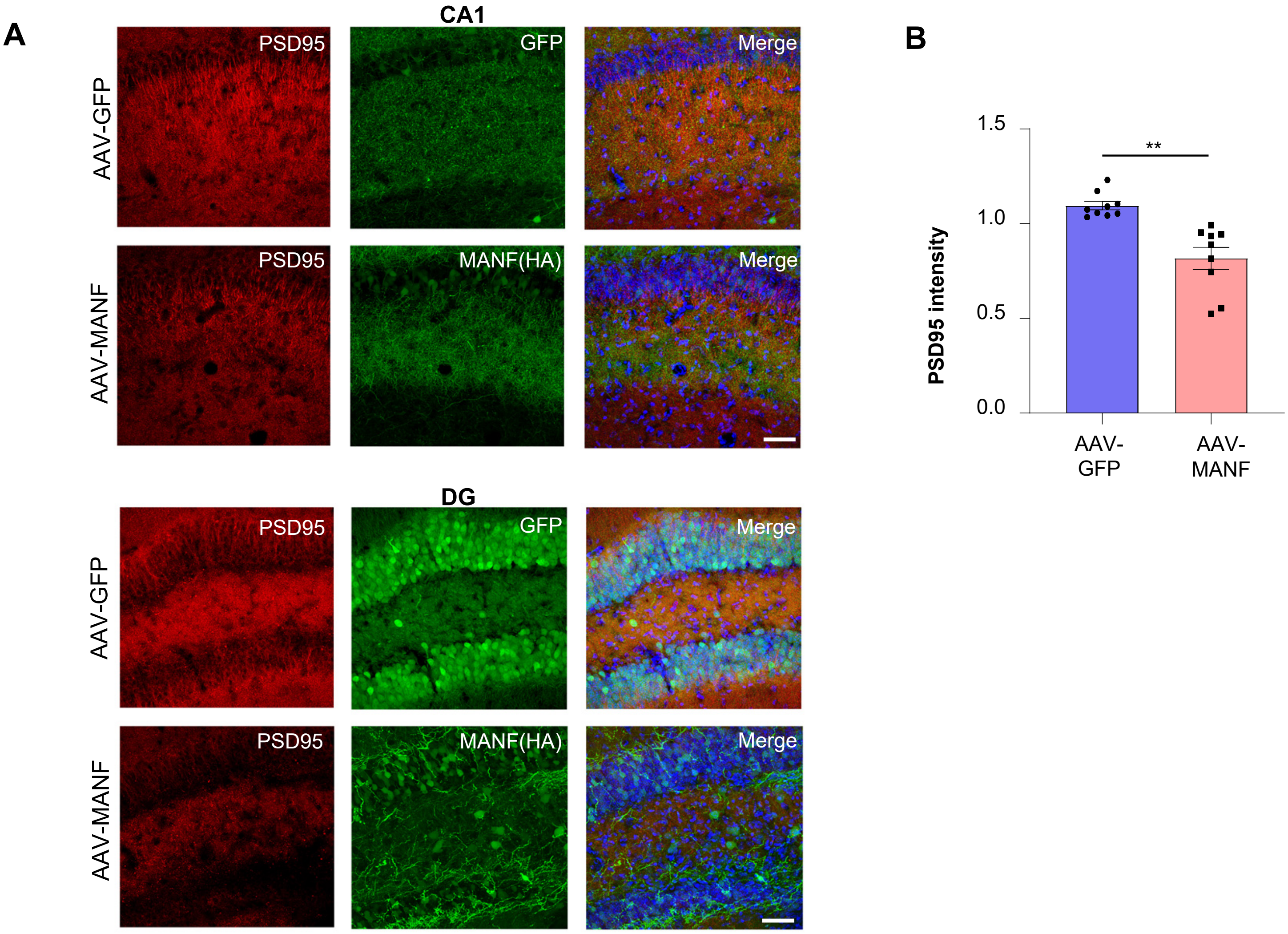

Figure S5. MANF overexpression in the hippocampus causes synapse loss (related to Figure 4). (A) Immunofluorescent staining images of PSD95 in the hippocampus of WT mice injected with AAV-GFP or AAV-MANF. GFP fluorescence and HA tag were used to indicate the expression of AAV-GFP and AAV-MANF respectively (scale bar: 50 μm). **(B)** Quantification of PSD95 staining intensity in the hippocampus of WT mice injected with AAV-GFP or AAV-MANF (n = 9 from 3 mice; two-tailed student t-test; *P* = 0.0025). ** *P* < 0.01. Data are represented as mean ± SEM.

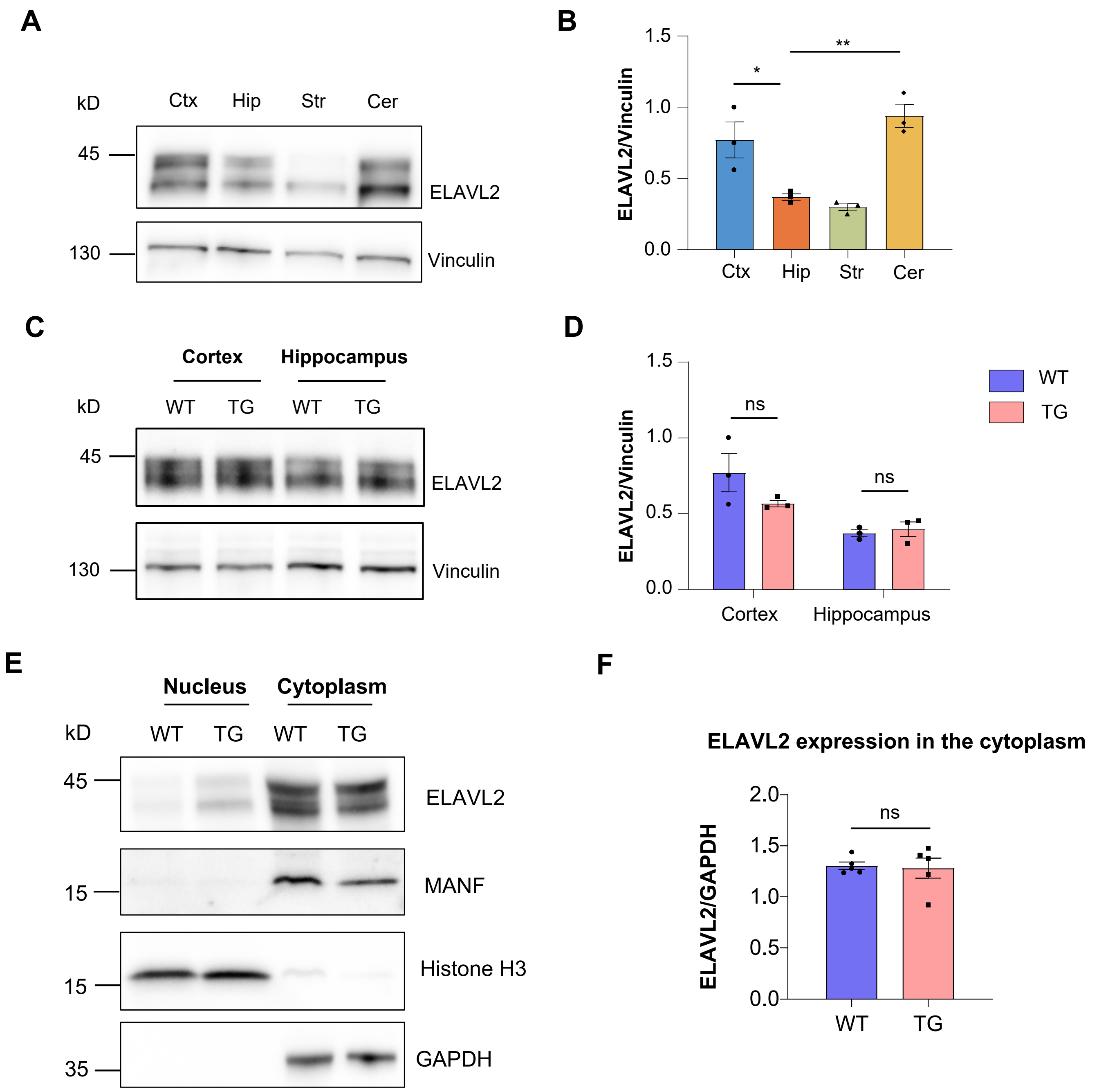

Figure S6. Characterization of ELAVL2 expression (related to Figure 5). (A) Representative western blotting images of ELAVL2 expression in different brain regions of WT mouse (Ctx, cortex; Hip, hippocampus; Str, striatum; Cer, cerebellum). Vinculin was used as a loading control. **(B)** Quantification of western blotting results in Fig. S6A (n = 3; one-way ANOVA with Tukey's multiple comparisons; Ctx vs Hip, *P* = 0.0269; Hip vs Cer, *P* = 0.0036). **(C)** Representative western blotting images of ELAVL2 expression in the cortex and hippocampus of WT and MANF TG mice. **(D)** Quantification of western blotting results in Fig. S6C (n = 3; two-tailed student t-test; cortex, *P* = 0.1908; hippocampus, *P* = 0.6452). **(E)** Representative western blotting images of ELAVL2 and MANF distribution in the nucleus and cytoplasm fractions from the brain of WT and MANF TG mice. Histone H3 was used as a marker for the nucleus, and GAPDH was used as a marker for the cytoplasm. **(F)** Quantification of western blotting results in Fig. S6E (n = 5; two-tailed student t-test; *P* = 0.8333). Ns, non-significant. Data are represented as mean ± SEM.

Figure S7

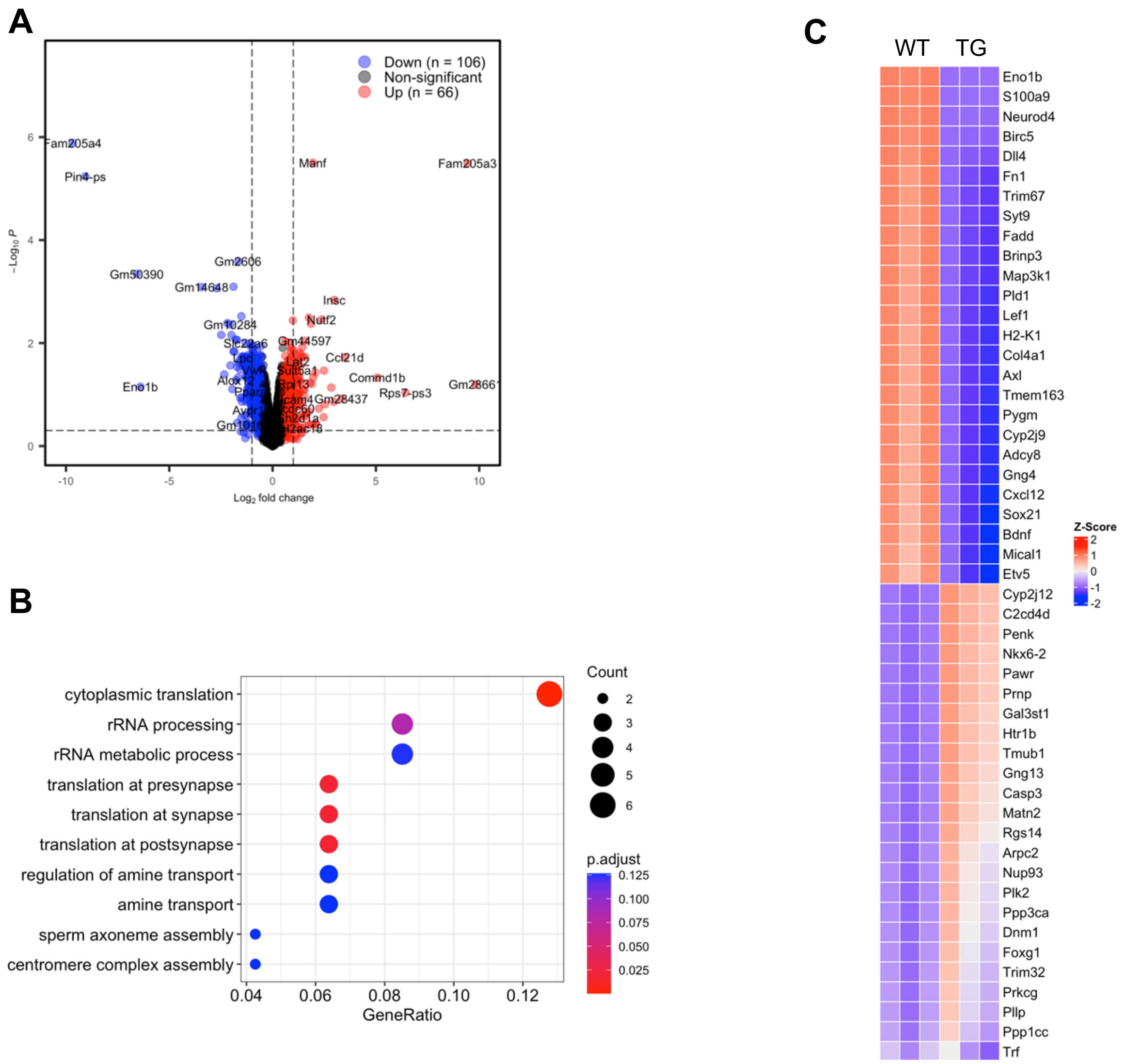

Figure S7. RNA sequencing analysis of the hippocampus from WT and MANF transgenic mice (related to Figure 5). (A) Volcano plot showing genes that are significantly upregulated (n = 66) and downregulated (n = 106) in the hippocampus of MANF TG mice, compared with WT mice. **(B)** GO pathway analysis of differentially expressed genes (DEGs). **(C)** Heatmap showing DEGs related to neuronal and synaptic functions when comparing WT mice with TG mice.

Figure S8

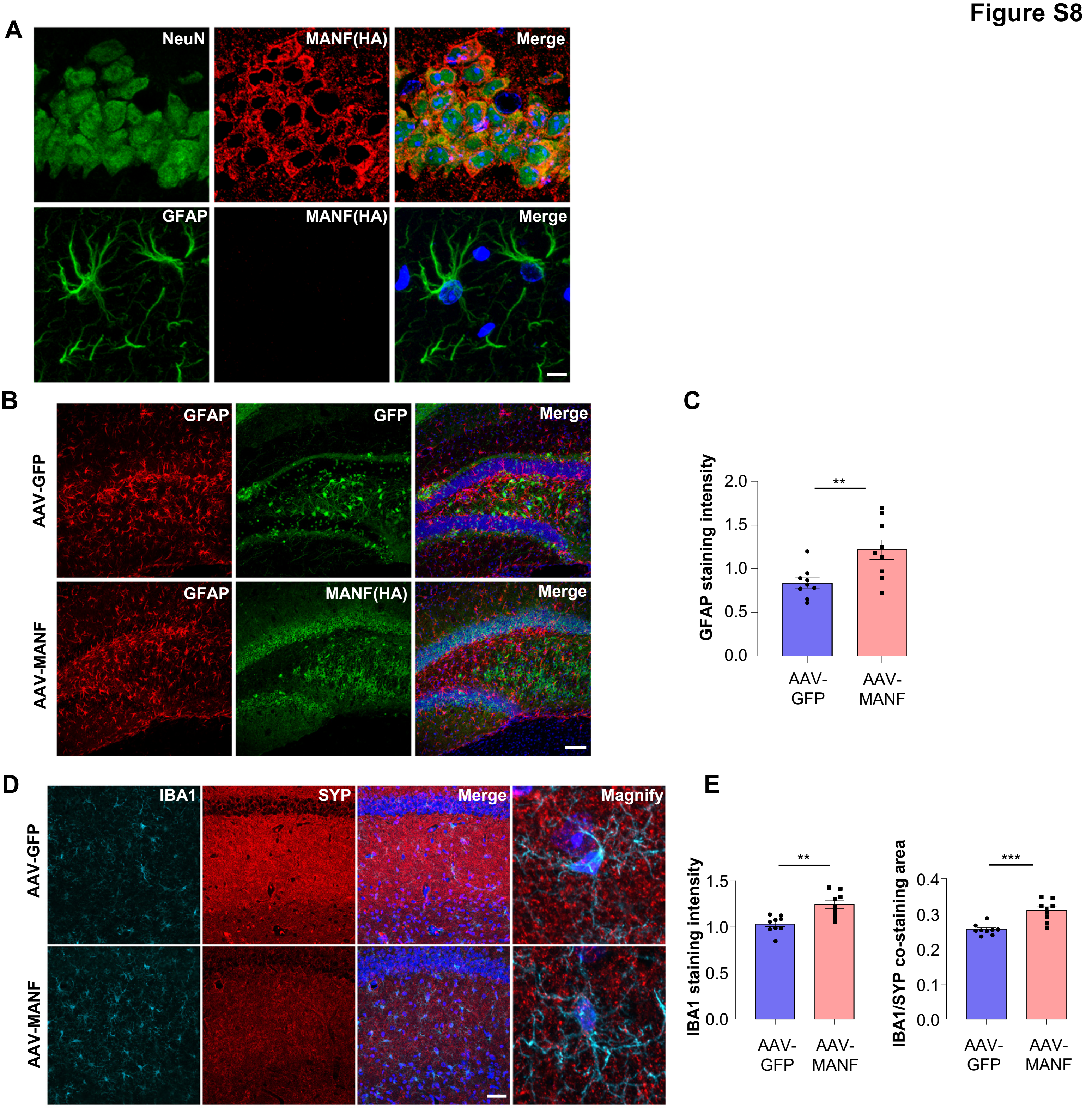

Figure S8. MANF overexpression aggravates glial activation in 5xFAD mice (related to Figure 6). (A) Double-immunofluorescent staining images of AAV-MANF and NeuN or GFAP in the hippocampus of 5xFAD mice (scale bar: 10 μm). **(B)** Immunostaining images of GFAP in the hippocampus of 5xFAD mice injected with AAV-GFP or AAV-MANF (scale bar: 100 μm). **(C)** Quantification of GFAP staining intensity (n = 9 from three mice; two-tailed student t-test; *P* = 0.0081). **(D)** Double-immunofluorescent staining images of IBA1 and synaptophysin (SYP) in the hippocampus of 5xFAD mice injected with AAV-GFP or AAV-MANF (scale bar: 50 μm). **(E)** Quantification of IBA1 staining intensity and IBA1/SYP co-staining intensity (n = 9 from three mice; two-tailed student t-test; IBA1, *P* = 0.0013; IBA1/SYP, *P* = 0.0002). * *P* < 0.05, ** *P* < 0.01. Data are represented as mean ± SEM.

Figure S9

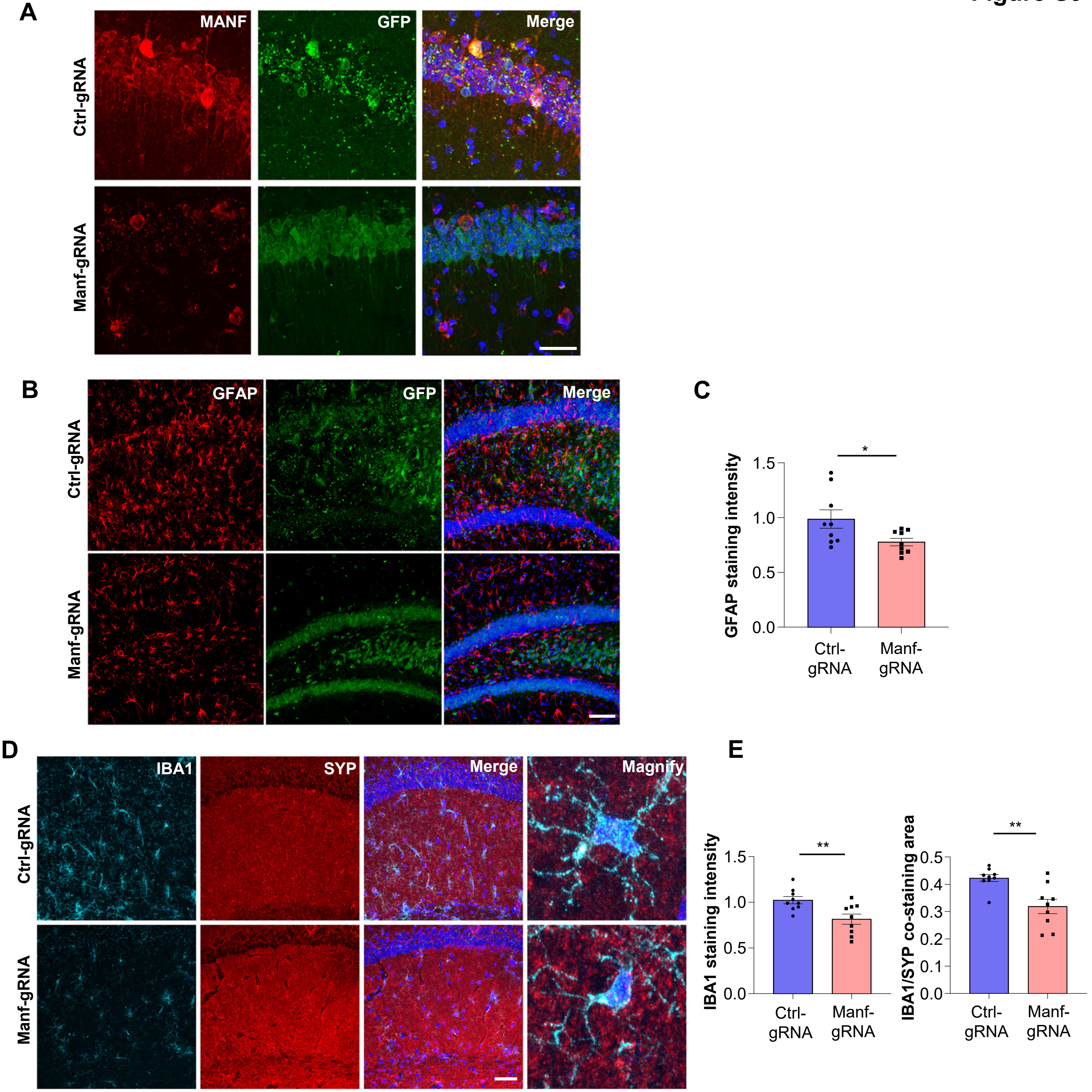

Figure S9. Reducing the expression of MANF alleviates glial activation in 5xFAD mice (related to Figure 7). **(A)** Immunostaining images of MANF in the hippocampus of 5xFAD mice injected with AAV-Ctrl-gRNA or AAV-Manf-gRNA. GFP fluorescence indicated the expression of gRNAs (scale bar: 50 μm). **(B)** Immunostaining images of GFAP in the hippocampus of 5xFAD mice injected with AAV-Ctrl-gRNA or AAV-Manf-gRNA (scale bar: 100 μm). **(C)** Quantification of GFAP staining intensity (n = 9 from three mice; two-tailed student t-test; *P* = 0.0333). **(D)** Double-immunofluorescent staining images of IBA1 and synaptophysin (SYP) in the hippocampus of 5xFAD mice injected with AAV-Ctrl-gRNA or AAV-Manf-gRNA (scale bar: 50 μm). **(E)** Quantification of IBA1 staining intensity and IBA1/SYP co-staining area (n = 9 from three mice; two-tailed student t-test; IBA1, *P* = 0.0084; IBA1/SYP, *P* = 0.0026). * *P* < 0.05, ** *P* < 0.01. Data are represented as mean ± SEM.
