# Supplementary material for: Increased expression of mesencephalic astrocyte-derived neurotrophic factor (MANF) contributes to synapse loss in Alzheimer’s disease
Source: Mol Neurodegener. 2024 Oct 18;19:75. doi: 10.1186/s13024-024-00771-3 (PMC11490049; doi:10.1186/s13024-024-00771-3)

Figure 1A Hippocampus 6M

MANF

25kD
15kD

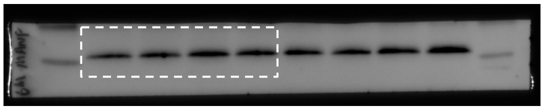

PSD95

180kD
130kD
100kD
70kD

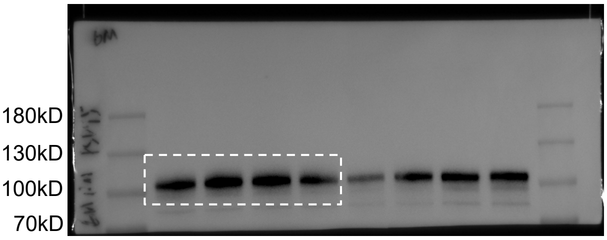

β-tubulin

70kD
55kD
40kD

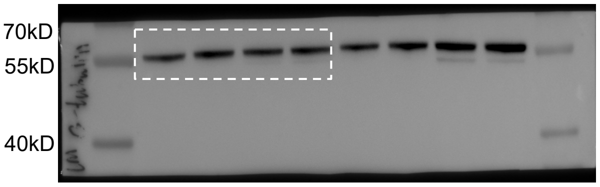

Figure 1A Hippocampus 12M

MANF

15kD

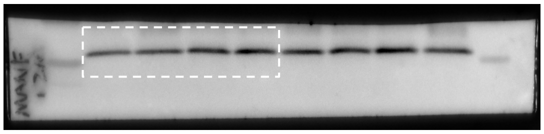

PSD95

180kD
130kD
100kD
70kD

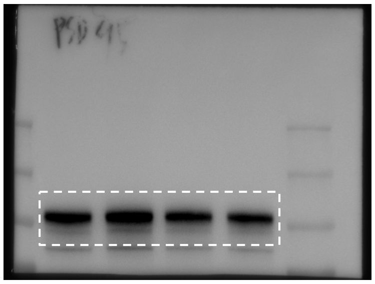

β-tubulin

70kD
55kD
40kD
35kD

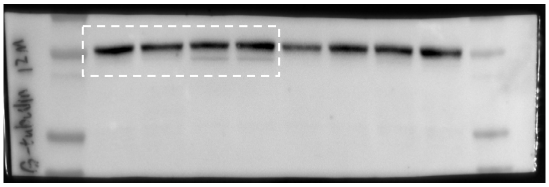

APP

180kD
130kD
100kD
70kD
55kD
40kD
35kD
25kD

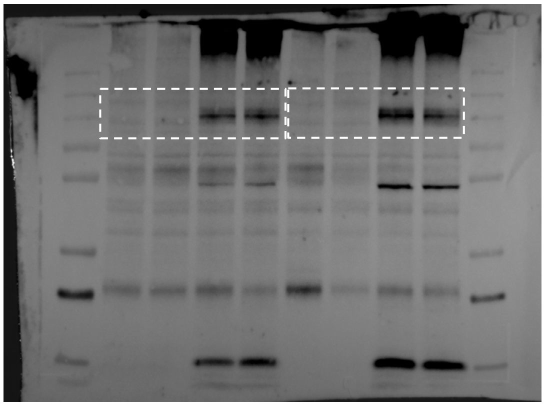

Uncropped images in Figure 1

Figure 1A Cortex 6M

MANF

25kD
15kD
10kD

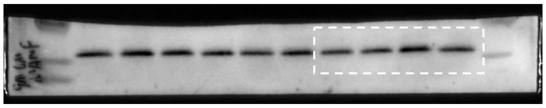

PSD95

180kD
130kD
100kD

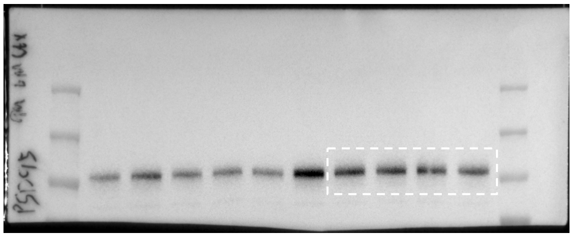

β-tubulin

70kD
55kD
40kD

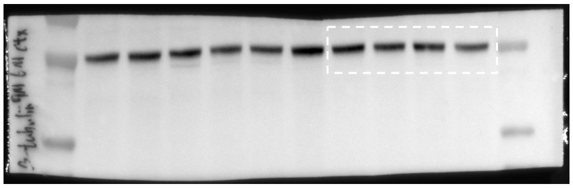

Figure 1A Cortex 12M

MANF

25kD
15kD

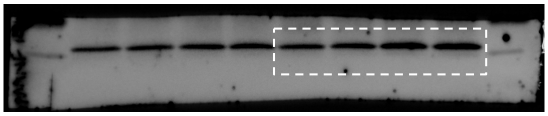

PSD95

180kD
130kD
100kD

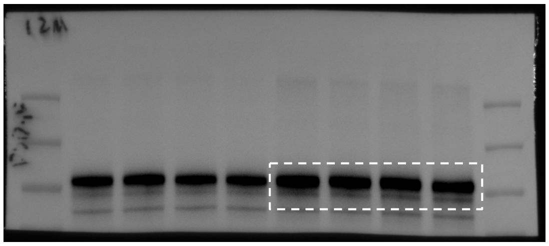

β-tubulin

70kD
55kD
40kD

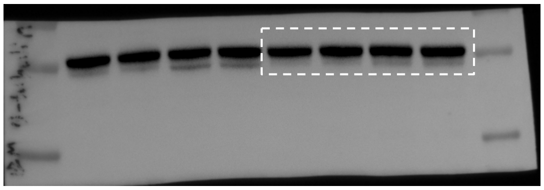

APP

180kD
130kD
100kD
70kD
55kD
40kD
35kD
25kD

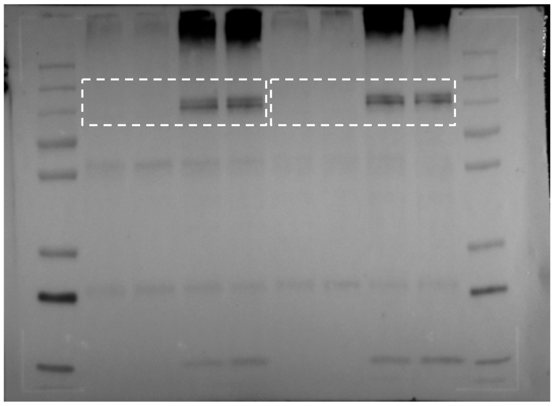

Figure 1E

MANF

25kD
15kD
10kD

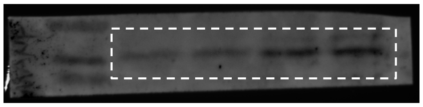

synaptophysin

75kD
65kD
45kD
35kD
25kD
15kD
10kD

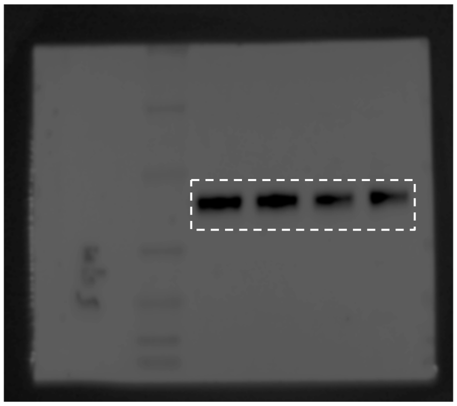

vinculin

180kD
135kD
100kD
75kD
65kD
45kD

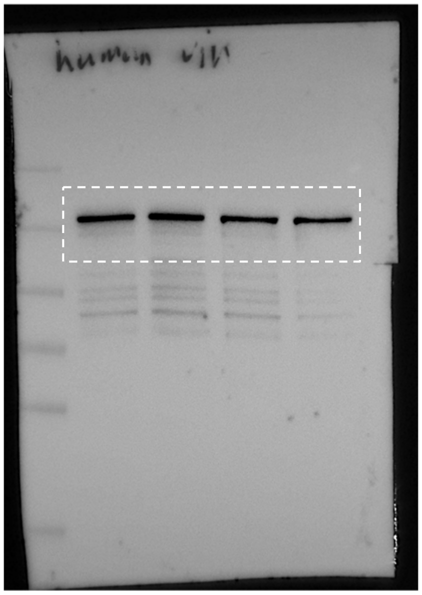

Figure 3A

PSD95

180kD
130kD
100kD
70kD

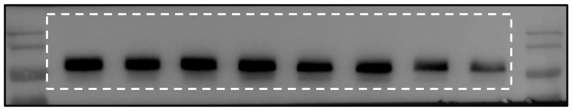

synaptophysin

40kD

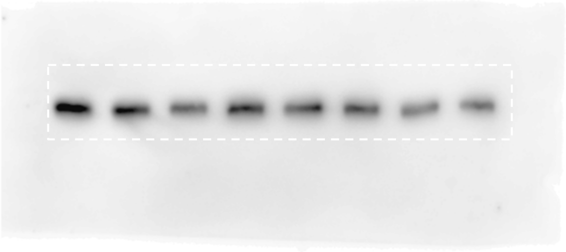

NeuN

65kD
45kD
35kD
25kD
15kD
10kD

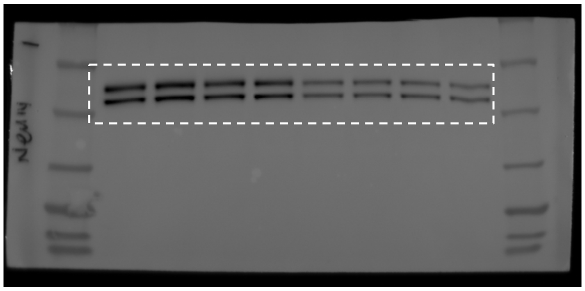

β-tubulin

70kD
55kD

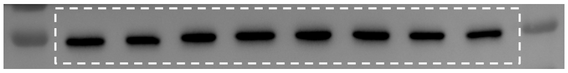

MANF-HA

35kD
25kD
15kD

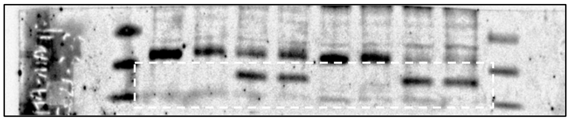

Figure 4G

PSD95

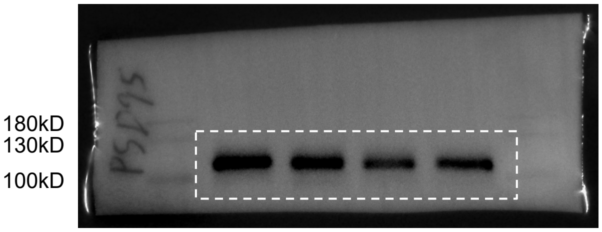

synaptophysin

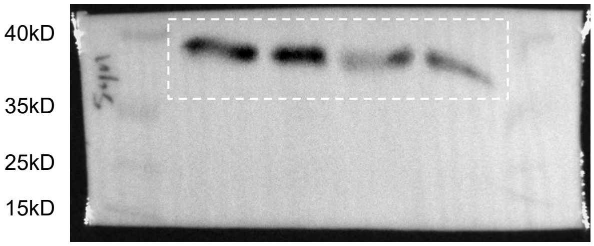

β-tubulin

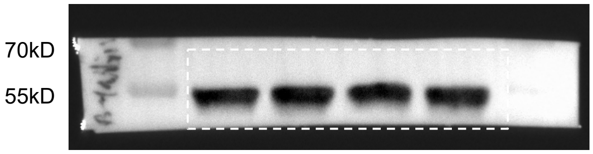

GFP

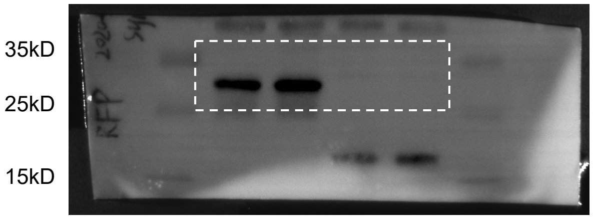

HA-MANF

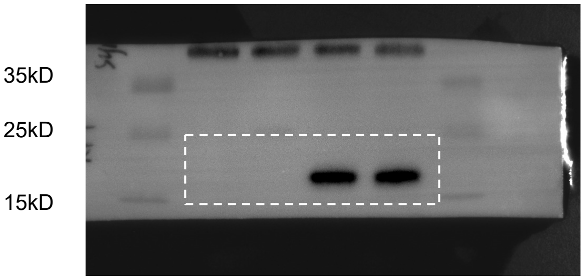

Figure 5A

MANF

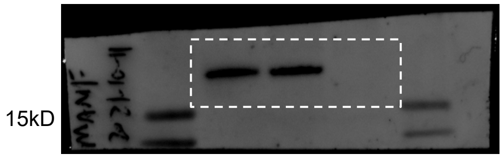

ELAVL2

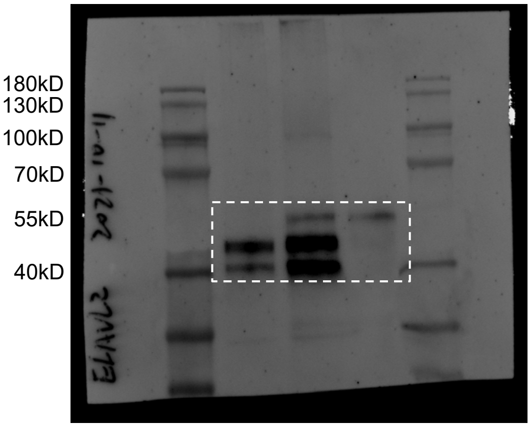

Figure 5B

GST-ELAVL2

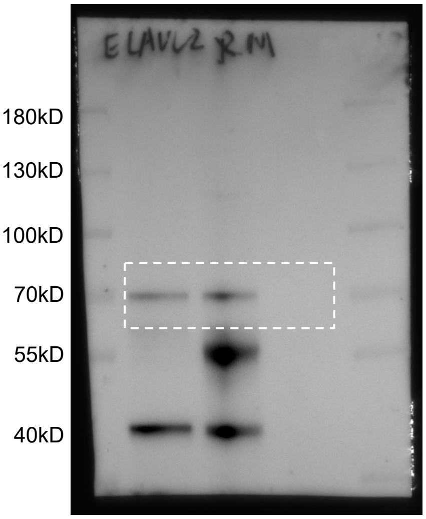

His-MANF

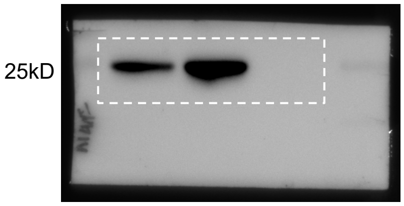

Figure 5D

His-MANF

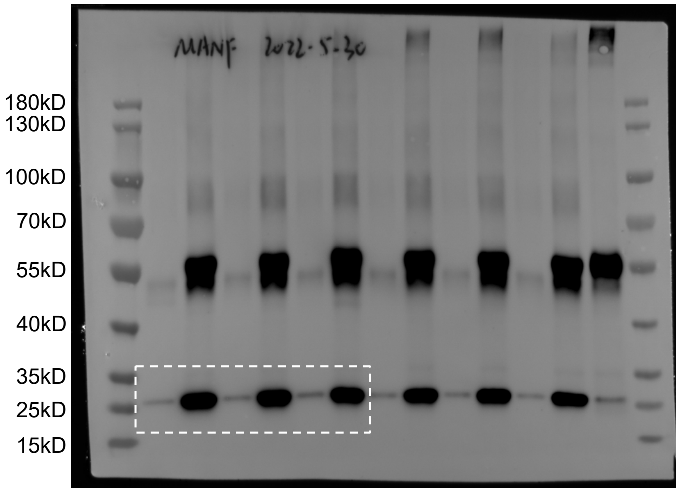

GST

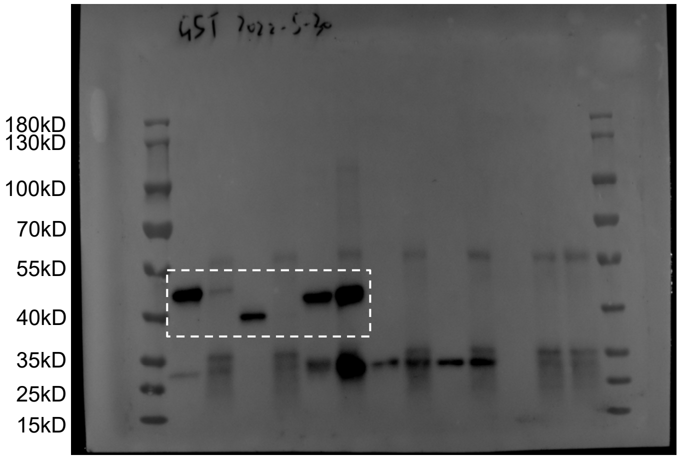

Figure 5G

ELAVL2

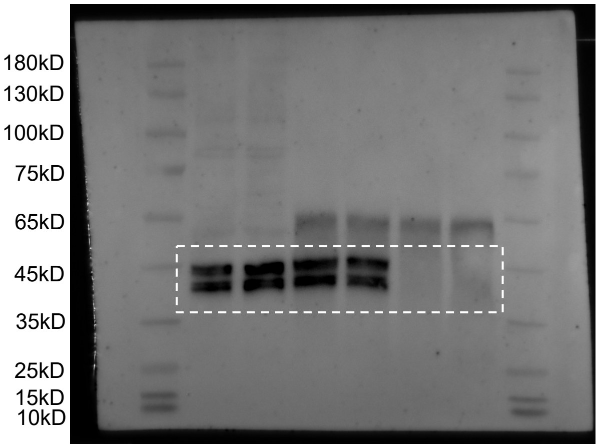

Figure 5H

Htr1b

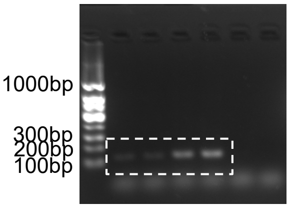

Bdnf

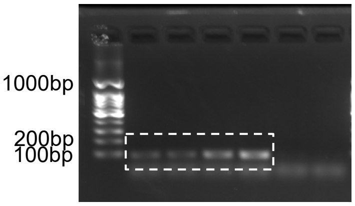

Casp3

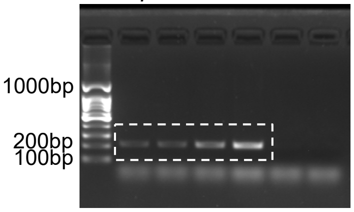

Adcy8

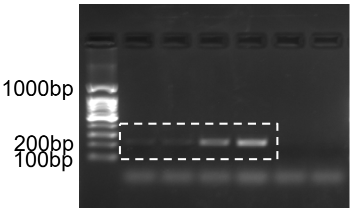

Uncropped images in Figure 6 & 7

Figure 6A

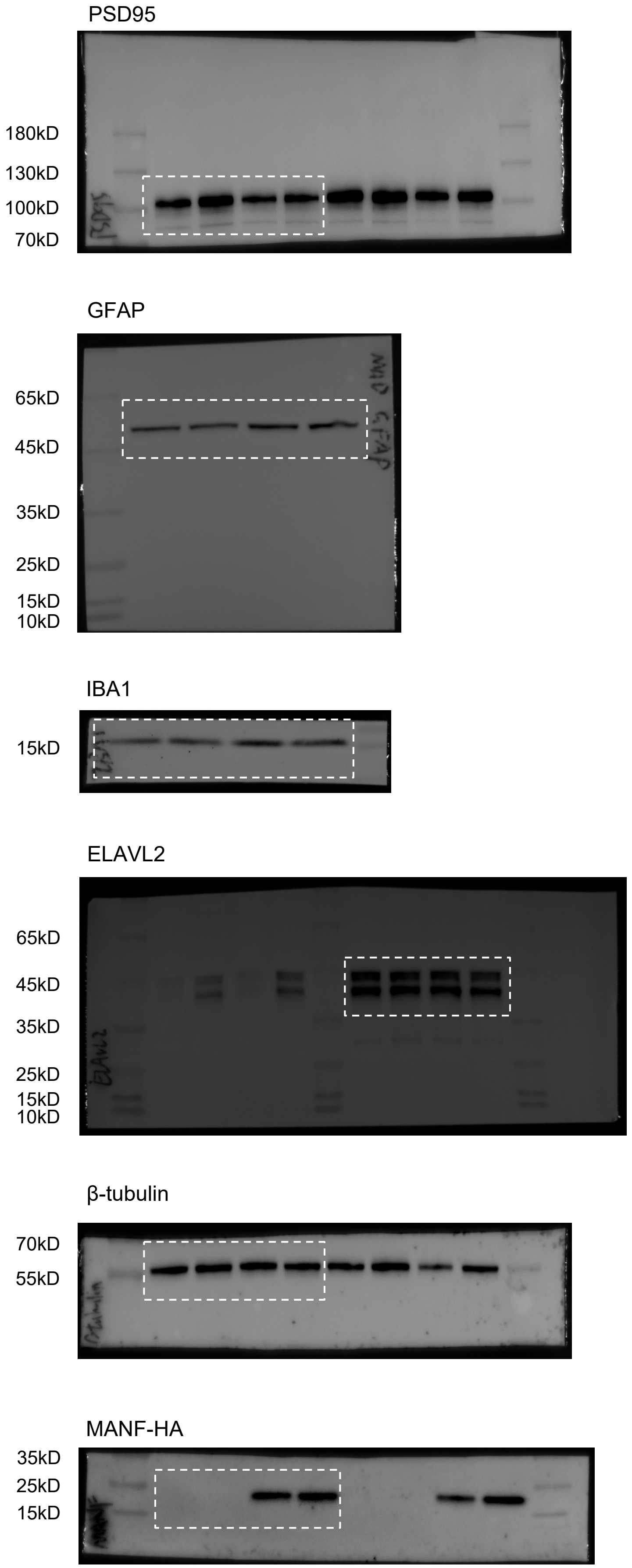

Figure 7I

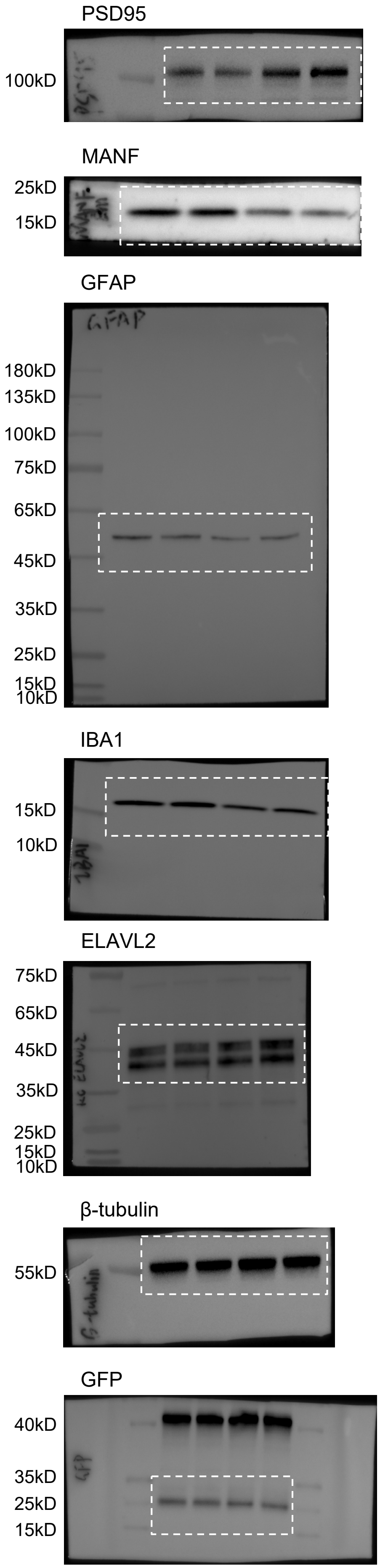

Uncropped images in Figure S1

Figure S1B 6M

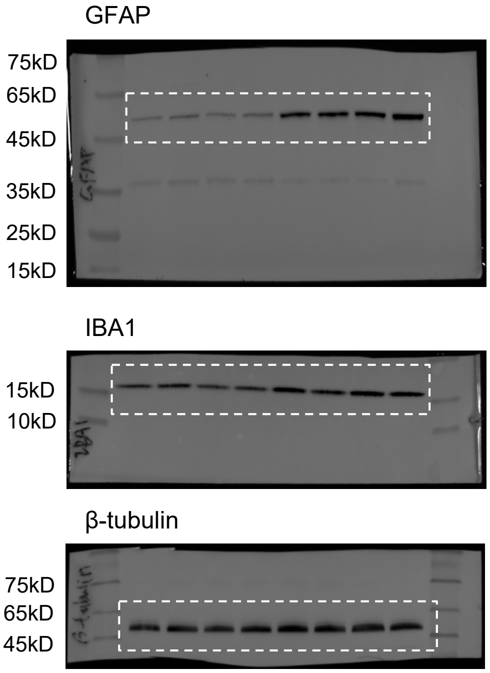

Figure S1B 12M

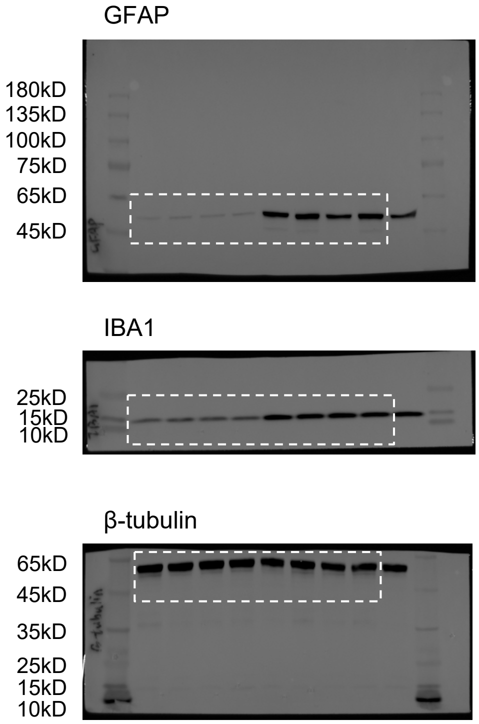

Figure S2C Hippocampus

MANF

25kD
15kD

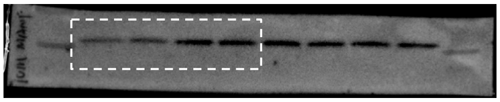

β-tubulin

70kD
55kD
40kD

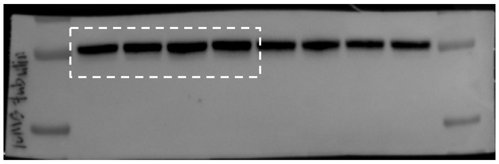

APP

180kD
130kD
100kD

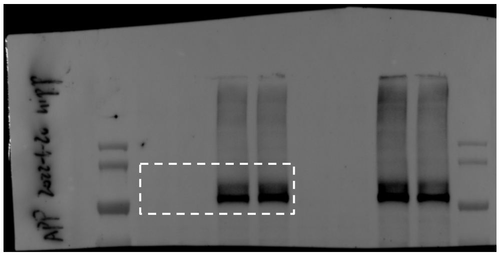

Figure S2C Cortex

MANF

15kD

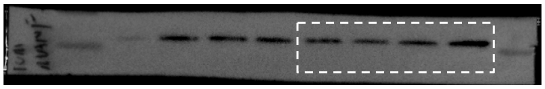

β-tubulin

70kD
55kD
40kD
35kD

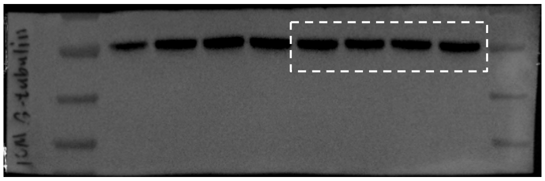

APP

180kD
130kD
100kD

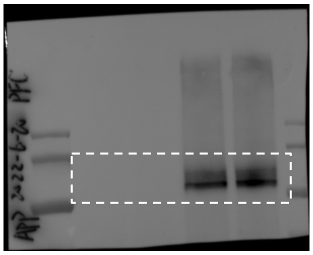

Figure S3A

GRIA1

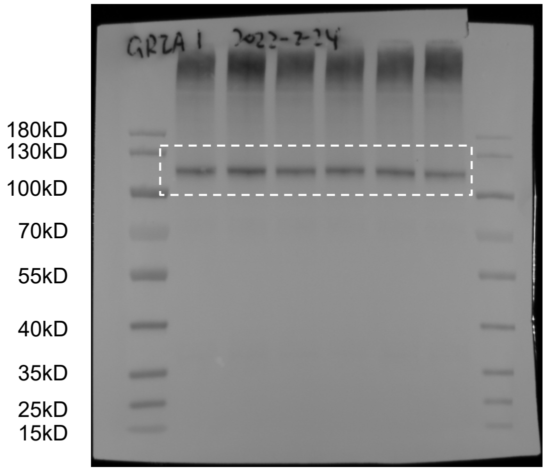

GRIN2A

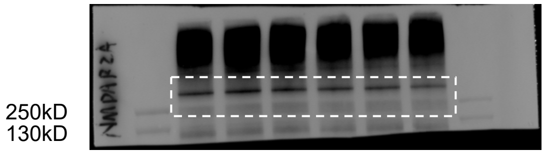

vinculin

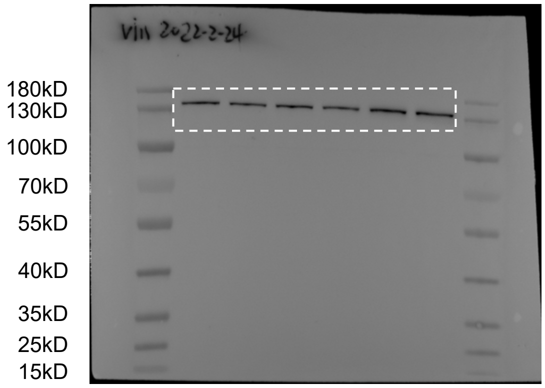

Uncropped images in Figure S6

Figure S6A

ELAVL2

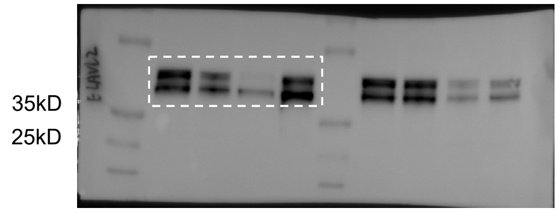

vinculin

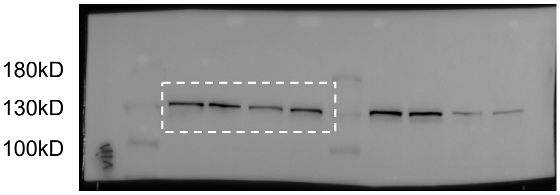

Figure S6C

ELAVL2

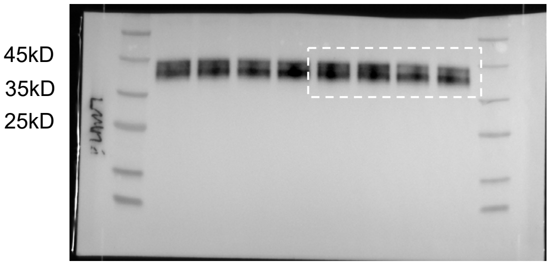

vinculin

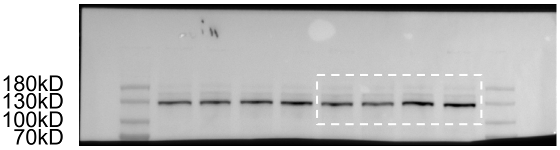

Figure S6E

ELAVL2

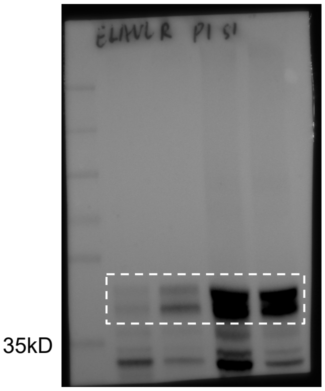

MANF

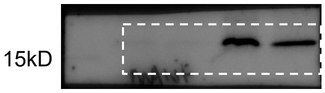

Histone H3

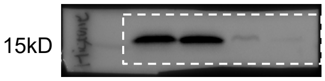

GAPDH

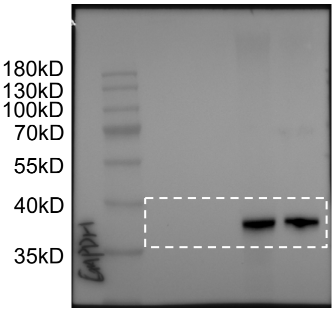

Supplement: Supplementary file 2 — Supplementary Material 2. [file 13024_2024_771_MOESM2_ESM.pdf]
